# An “Arcipilago” as an Intellectual Ecosystem: In Search of Jean Piaget’s Library Through a Visual History

**DOI:** 10.1007/s42087-025-00475-0

**Published:** 2025-02-17

**Authors:** Marc J. Ratcliff

**Affiliations:** Centre Jean Piaget – FPSE – University of Geneva, Geneva, Switzerland

**Keywords:** Jean Piaget, Library, Piaget archives, Piaget’s office, Visual history

## Abstract

Divided into three parts, this article highlights the spatial and temporal boundaries of Jean Piaget’s library through a visual history of his office based on photographs taken since the late 1960s. First, we showed how Piaget’s library, as part of an intellectual ecosystem, sedimented along a spatial boundary into a horizontalized space, the “archipilago,” consisting of an archipelago of book stacks and files, and a vertical space, the wall-mounted bookcases. This division of space corresponded to two donations of works to the Piaget Archives made at different times. It also highlights the fact that Piaget’s writing space became *mobile* at a certain point in time, which has yet to be determined. Second, we scrutinized Piaget’s intellectual ecosystem, asking how his disorder was structured and what its temporal boundaries were. To answer this question, we developed a model that distinguished between two types of behavior specific to an intellectual ecosystem: research behavior and archival behavior, the former managing the potentiality of the elements it processed (open segments), while the latter closed many of these segments. Applying this model, it becomes clear that certain areas of the office were closed at a certain point in time, especially the library, which was thus partially inaccessible. The third part focused on the heuristic perimeter of this intellectual ecosystem, using statistical analyses of the library to complement its visual history. These analyses allowed us to better understand Piaget’s relationship with certain issues, such as the time it took him to build up his library, the types of reading he did, the evolution of his relationship with interdisciplinarity and the interdisciplinary perception of him by the scientific community, and the geographical distribution of the works dedicated to him at different times. The article concludes by showing how the analysis of the library allowed us to date the period when the writing space became mobile, Piaget responding to changes in his intellectual environment by modifying the shape of his ecosystem.

## Introduction

This article brings together two fields of study, Piagetian historiography and library historiography, using a dual methodology (visual history and statistics) to problematize a model of the researcher’s relationship with his or her work, with particular reference to the problem of order and disorder.

From the long history of libraries (Carbone, 2012; Barbier, 2013), we won’t be dealing with the library as a place of knowledge with a collective vocation (Jacob, 2001, 2014), but rather with an author’s library (Quarto, 2010; Belin, Mayaux & Verdure-Mary, 2018; Fumaroli, 2021) and, moreover, with an author who is difficult to classify because he is endowed with multiple identities. Research on author and, especially, writer libraries has opened up many windows on the author and his multiple extensions. For example, they have contributed to an author’s biography by reconstructing his or her sociabilities, using collections of books, sometimes with dedication. These are the surrealist and resistant sociabilities of a poet such as Paul Eluard (Belin, 2018). The study of writer’s libraries, seen as a working tool (Cudré-Mauroux, 2018), has also enriched genetic criticism and the explanation of literary creation (D’Iorio & Ferrer, 2001), for example by analysing reading practices, as in the case of Marguerite Yourcenar’s library (Chehab, 2018). Some authors placed the experience of reading among the fundamental experiences of the writer (Ouvry-Vial, 2007: 167).

The question of order is often raised in the historiography of libraries. Christian Jacob argued that order is inherent to the library, which, “like human memory, needs order to function” (Jacob, 2001: 66). Order means classifiying, by discipline, by field, by subject, a shared and comprehensible classification. It is therefore a boundary between public and private libraries, since in the latter the order can be sometime not intended to *be understood by everyone*, unlike the shared classification of public libraries. Of course, some authors adopted a transparent order such as the thematic order in the library of the Polish poet Herbert (Citko, 2018) or the “classification by field” in Jean Starobinski’s library (Reichler, 2010). In fact, every researcher is confronted with the question of disorder and order, and this equation hasn’t really changed with the transition to an immaterial, virtual world. This is one of the issues that this paper is concerned with: in the author’s library, it’s as much a question of understanding his or her order - how is the order structured - as it is of asking under what conditions is this order comprehensible? Hence we’ll look at the researcher’s relationship to his or her (dis)order, both diachronically and synchronically.

These questions, and disorder in particular, have only recently been addressed in Piagetian historiography. Since the 1990s, Piagetian historiography has been primarily concerned with the history of ideas. It has offered overviews of Piaget’s work in collectives (Müller et al., 2009), intellectual biographies (Ducret, 1990; Vidal, 1994; Kohler, 2008), epistemological (García, 2000; Richelle, 2000), methodological (Bond & Tryphon, 2009) or historical (Perret-Clermont & Barrelet, 2008) approaches. The criterion common to this historiography can be reduced to the interpretation of Piagetian theory, divided along an axis in which history and epistemology are the two poles (Ratcliff, 2024).

It is a recent dissertation (Noël, 2020) on the public image of Jean Piaget that has helped to renew the historiographical approach by showing that the office and disorder became constitutive features of Piaget’s public persona, which from the late 1960s was “built around a verbal and visual trilogy: that of the pipe, the bicycle and the office” (Noël, 2020: 295). The study showed that his office acquired its iconic meaning in the context of media and public sphere. Facing the media context, Piaget also made it a rule, which Noël called the biographical injunction, not to reveal anything about his private life unless it related to his work. It was within this scope that the question of disorder was raised: in 1969, when the journalist Jean-Claude Bringuier diplomatically uttered the word “disorder”, Piaget laughed and replied: “As you know, Bergson showed that there is no disorder! There are two kinds of order: geometric order and vital order. Mine is clearly vital” (Bringuier, 1977: 15). This response has gone down in history as “vital disorder”, or how to share a disorder that is fundamentally opaque to observers. However, it was also within this same media framework that the question of how Piaget functioned in his office was answered in small ways. In other interviews, Piaget would add that he had “a sense of order that helps (him) find everything” (Noël, 2020: 276), that “the files I need are at my fingertips, in order of frequency…” (Bringuier, 1977: 15), or that “every book is arranged in chronological order - even those under coats and blankets on tables next to crowded shelves” (Noël, 2020: 275). Piaget’s emphasis on the temporal dimension - order of frequency, chronological order - is noteworthy.

These questions are echoed in the historiography of libraries. In the case of Starobinski, Stéphanie Cudré-Mauroux drew on the arts of memory to understand how the researcher functioned in his interior: She “found each of his books (…) thanks to the visual combinations and the location of the books in spaces that each represented one of the places in the critic’s vast field of interest.” (Cudré-Mauroux, 2018). This transparent classification was based on a mental cartography whose temporal dimension was that of the trajectories prepared by the classification, understandable to the observer. But what happens in the case of a non-transparent classification, as it is with Piaget, working in a ‘disorder’ for the observer? How are the relationships between material books and projects managed, and according to what spatial and temporal boundaries? To answer these questions, we’ll develop a model to help us understand the complexity of the researcher’s relationship with his disorder, a model derived from previous research (Ratcliff, 2015) that took into account the question of temporal boundaries in the case of the researcher.

Methodologically, we will approach these questions through two methods rarely combined. On the one hand, we will analyse a series of iconographic sources of photographs, taken during interviews with Piaget between 1969 and 1978, comparing them with images from a photographic campaign of the office in the summer of 2012, carried out by the Piaget Archives. On the other hand, libraries contain serial data, i.e. series of works, which deserve to be analysed using statistical tools, all the more so if, as we shall see, we can proceed by relevant zoning of the library.

## I. Looking for Piaget’s Library: A Visual History

**Fig. 1:**
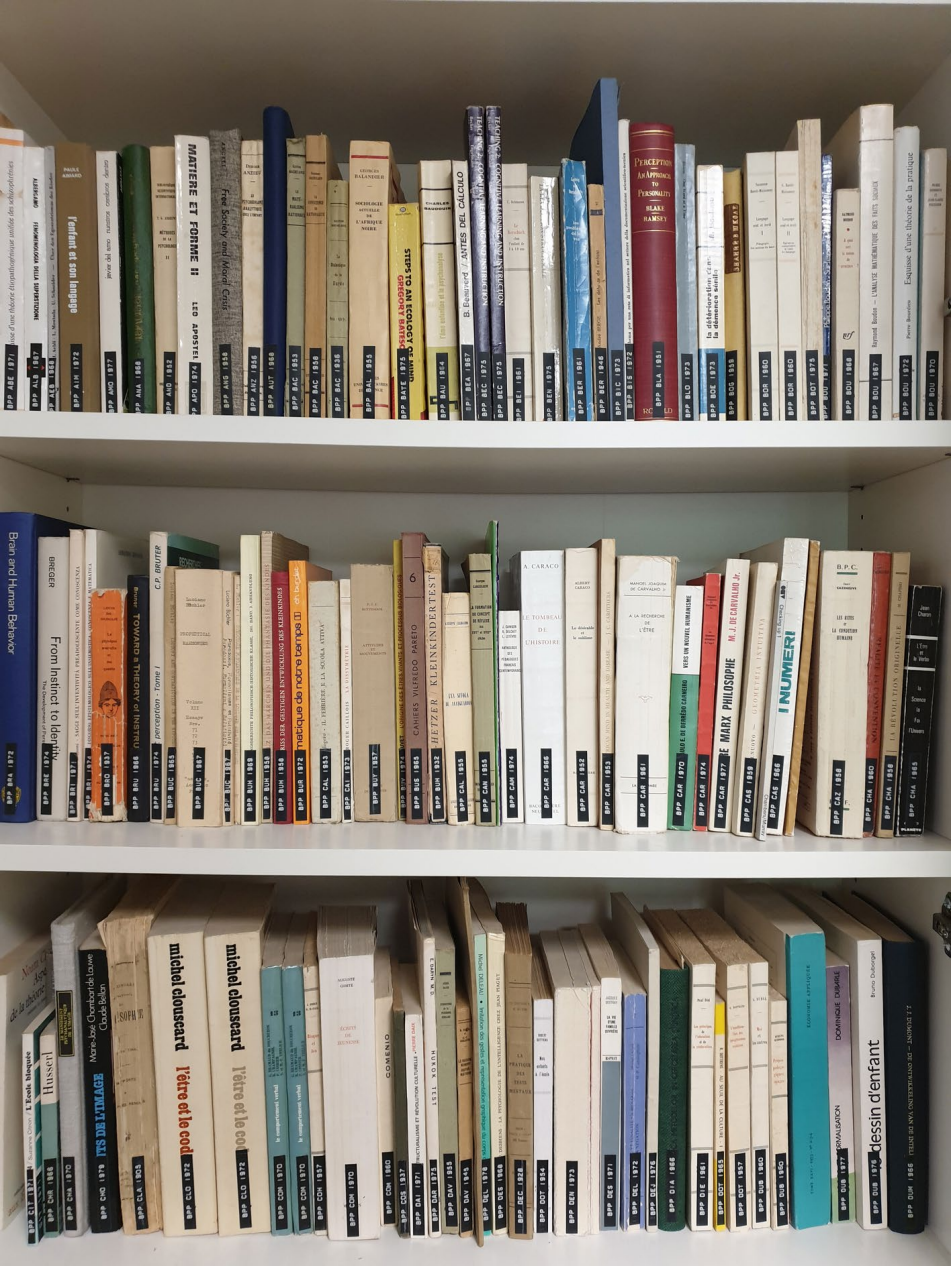
Part of Jean Piaget’s library (BPP) at the Centre Jean Piaget. © Centre Jean Piaget

**Fig. 2:**
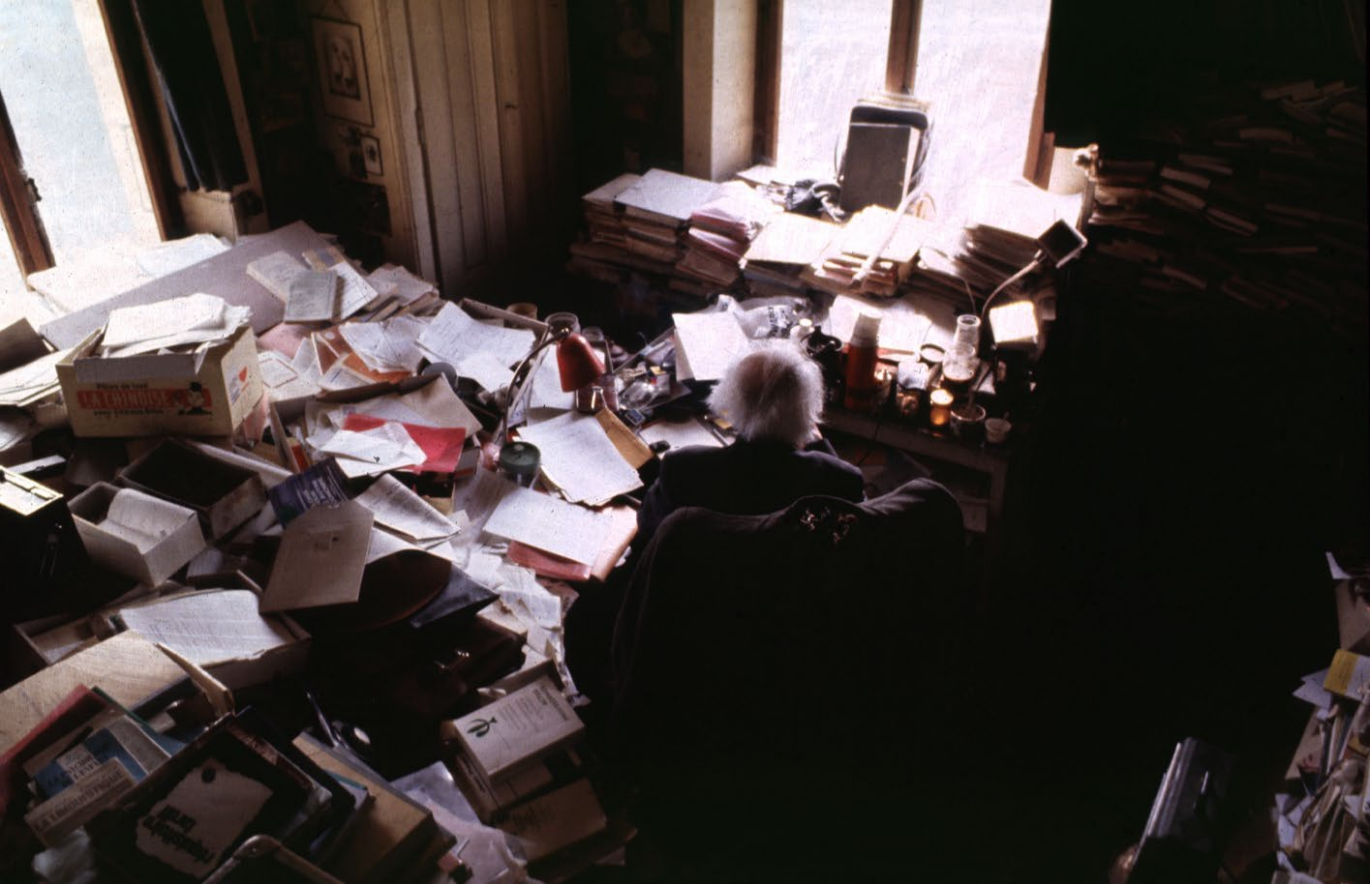
Photo of Jean Piaget’s office, ca. 1978, © Archives Jean Piaget, unknown photographer

If you ask what Jean Piaget’s library was, you’re faced with a Cornelian choice: is the concept conveyed by this image (Fig. 1) or this one (Fig. 2)?

As we can see, on the one hand (Fig. 1), his library was indeed… a library! In other words, a collection of monographs that once belonged to a private individual, removed from their place of residence, and subjected to library good practices: cataloging, listing, shelving in a certain order, and then making them available to the public via a website. On the other hand, the second image shows Piaget’s office, an iconic image that has become a symbol of external disorder masking internal order.

Which of the two is the “real” library? The answer, of course, is historical, genetic: these two photographs correspond to two different moments in time, as well as to two states of a similar whole. The aim of this article is to reconstruct this genesis and to put the life of a library into context through a visual and statistical history. But it is not without raising a number of questions: is the right unit for thinking about the life of a library the library itself, or it is a part of an intellectual ecosystem? Where are the boundaries?

### Archival Boundaries

The transfer of an individual’s library to an institution, which requires numerous operations such as sorting, classification, selection (by author, by family), is generally accompanied by the loss of the author’s order––but also of his/her disorder!––as well as the many spatial, temporal, and semantic boundaries that created the dynamics of this ecosystem. We’ll ask ourselves how we can think about boundaries and limits within the framework created by the author.

We can distinguish between semantic objects and functional objects. Semantic objects include all forms of archives with meaning: manuscripts, notes, correspondence, notebooks, administrative documents, research data, printed matter, etc. Functional objects include furniture (shelves, desks, tables, chairs) and work tools (storage units, filing cabinets, pens, ink, paper, etc.). Then it appears that the library is the result of an artificial gesture that consists in grouping all the monographs together––on the basis of their common format––to form the library, yet *isolating it* from its intellectual ecosystem. What does this mean? To understand it, we need to look at the history of this ecosystem. This story includes the donations made to the Piaget Archives, which not only transformed certain elements of a private ecosystem into a public object, but also made it possible to reconstruct its history.

### Two Donations to the Jean Piaget Archives

The history of these two donations is very uneven, the first having been made almost without trace, while the second was made more recently and was controlled by the Piaget Archives.

The first donation dates back to the mid-1990s. About fifteen years after Piaget’s death in 1980, his son Laurent decided to donate some of his father’s books to the Jean Piaget Archives. The date of the donation is uncertain, but it probably took place in 1996, on the occasion of the centenary of his father’s birth. This donation consisted of books only; it was catalogued and bears the reference BPP for *Bibliothèque Privée Piaget*. The second donation is from 2012. In 2011, Laurent Piaget, who lived in family house, died, and after a meeting with Didier Morier, the representative of the family, the idea of an agreement to protect and preserve the intellectual content of the house Piaget was born. The agreement was signed in May 2012 between the Piaget Archives and Jean and Valentine Piaget’s two daughters, Jacqueline and Lucienne. Through this agreement, the Piaget family donated to the Archives Piaget all the intellectual content of the Villa Piaget, including the library. The sorting, selection and collection of this *Piaget Family Donation* (DFP) of almost 80’000 documents was done between 2012 and 2018. A grant from a private foundation in Geneva was obtained for the cataloging, inventory and partial digitization of the collection.

However, if our sorting was based on controlled criteria––for example, the almost systematic restitution of the material provenance of the archives and works in the office and in the house––this was not the case for the first deposit, the sorting criteria of which we do not know. We can, however, try to reconstruct them through a visual history of the first deposit around 1996.

### The First Deposit Circa 1996 : A Visual History

**Fig. 3:**
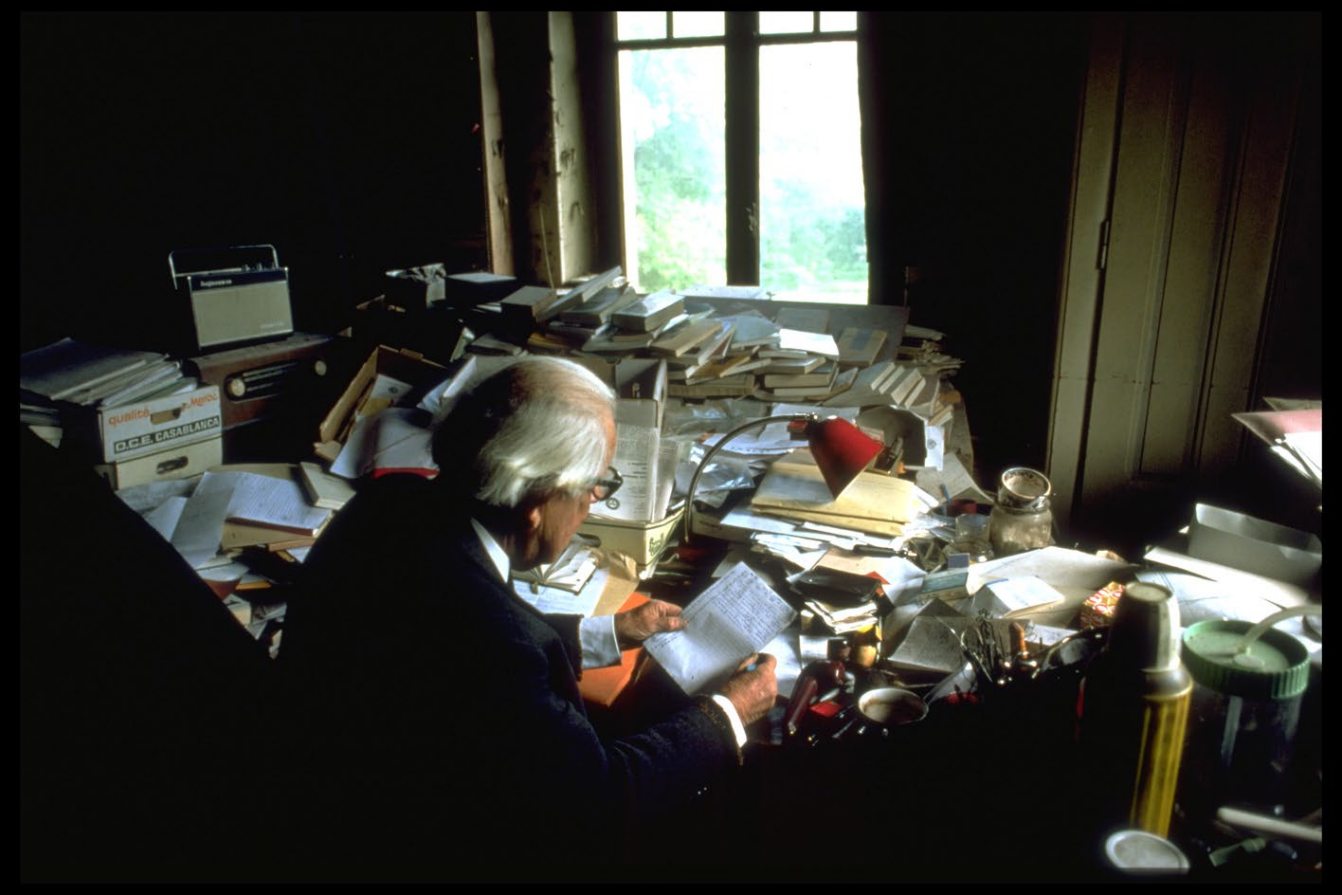
Stacks of books and files in front of the south office window, circa 1974. © Archives Jean Piaget, unknown photographer

**Fig. 4:**
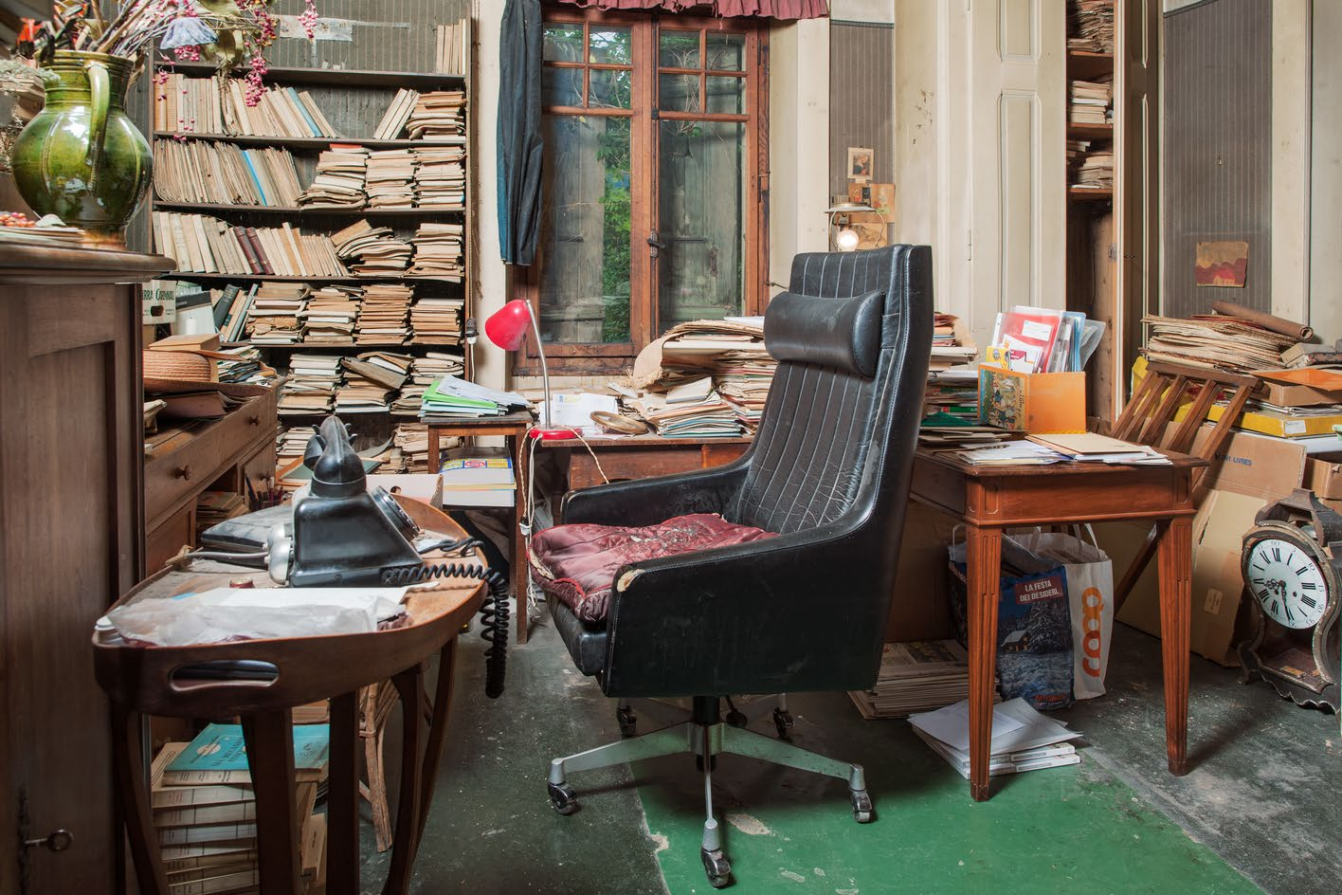
The same location in 2012, seen from the left: only piles of files remained. © Archives Jean Piaget, Photo R. Chappellu and D. Ponté

Laurent’s deposit consisted of 550 books. Of course, this was not the entire Piaget library, but a partial donation. But how was it sorted? What were Laurent’s selection criteria? We can answer this question thanks to a series of photos of Piaget’s office taken in the 1970s and compared with those of the photographic campaign we carried out when we entered the villa in 2012. The 2012 photos show that all the bookshelves in the office were practically everywhere full of books, and their arrangement was often similar to the 1970s photos. Therefore, these 550 books came from somewhere else, and the only explanation was that they were in piles, as this comparison of two photos from the 1970s (Figs. 3) and 2012 (Fig. 4) shows.

We can therefore answer the question of what the selection criteria were: the aim was to remove books from the numerous stacks (Fig. 3) that were blocking circulation in the office, and this opens a window on Laurent’s motivation. It seems that the first deposit was simply the result of his effort to tidy up the office, and therefore to remove the books from the piles that had been placed on tables and obstructed the passage. This is evidenced by the fact that in 2012 many stacks *of files* were still present on the various tables (Fig. 4), but no longer obstructing the passage. And the numerous tables and shelves in the office (no fewer than 8) formed the backbone on which the original stacks rested.

So, in the spirit of tidying up and “normalizing” the office, Laurent emptied the circulation space and intervened with the stacks. There were indeed Piaget stacks throughout the office, but these stacks were not just piles of books: rather, they were an “archipilago” or archipelago of piles made up of stacks that combined books and specific research files. This seems to be the way Piaget worked, at least in the 1970s, as shown in photos 5 and 6.

**Fig. 5:**
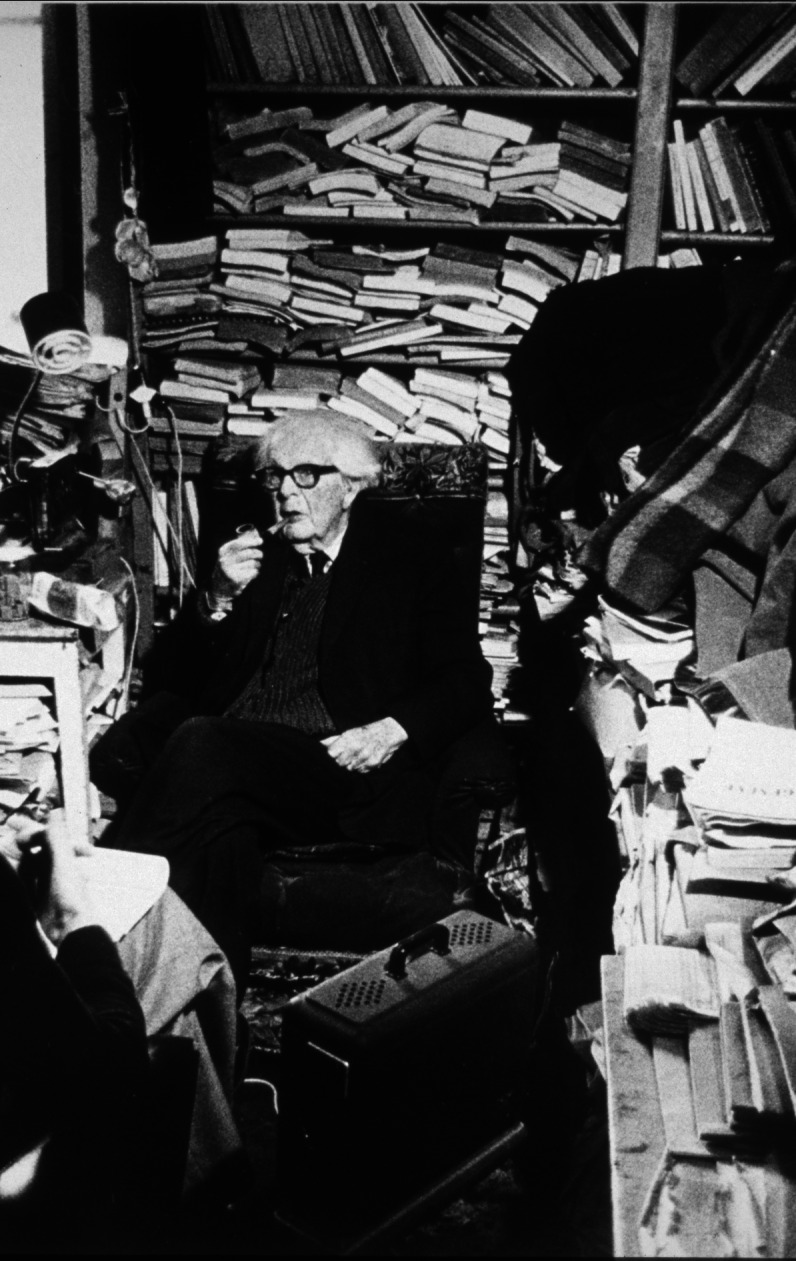
An archipilago element: books stacked in the library, c. 1974. © Archives Jean Piaget, unknown photographer

**Fig. 6:**
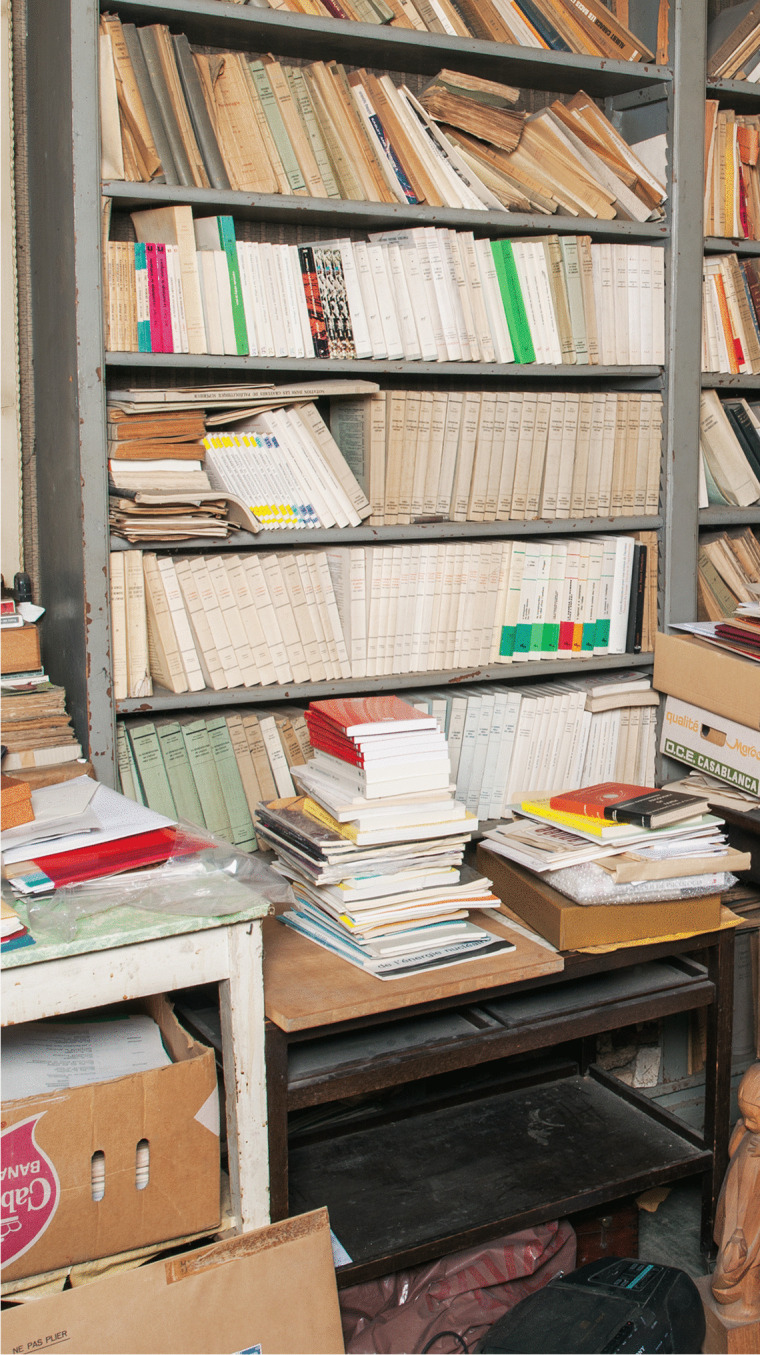
The same space ‘librarianized’ by Laurent Piaget. © Archives Jean Piaget, Photo R. Chappellu and D. Ponté

In Fig. 5 from 1970, we see shelves filled with books stacked horizontally; the Fig. 6 shows the same space in 2012, ‘librarianized’ by Laurent, who had arranged doublets of his father’s works.

As a result, Laurent’s approach to the office significantly altered his father’s (dis)order, including the physical parts (shelves) of the library where Piaget had stacked books. By tackling the stacks of mixed files and books, and the horizontal books *rather than the vertical books* on the shelves, Laurent constructed a different “surface” order, achieved mainly by separating the stacked books from the files. However, the destination of each, books and files, was different: separated from the works that concerned them, the files were kept in the office, placed in boxes or stacked on a few tables, while the books were collected to form the first donation.

Laurent’s clearing operations also revealed elements of the office’s stratigraphy, in particular the fact that Piaget’s writing area, the place at which he worked, photographed in the 1970s, must be seen as part of a genealogy, that is, its *ancestor* was an earlier piece of furniture that was, in a sense, buried by what followed. This can be demonstrated by marking the spaces in the following images, bearing in mind that Piaget works was the center of the archipilago in order to move as little as possible to easily reach the current elements.

**Fig. 7:**
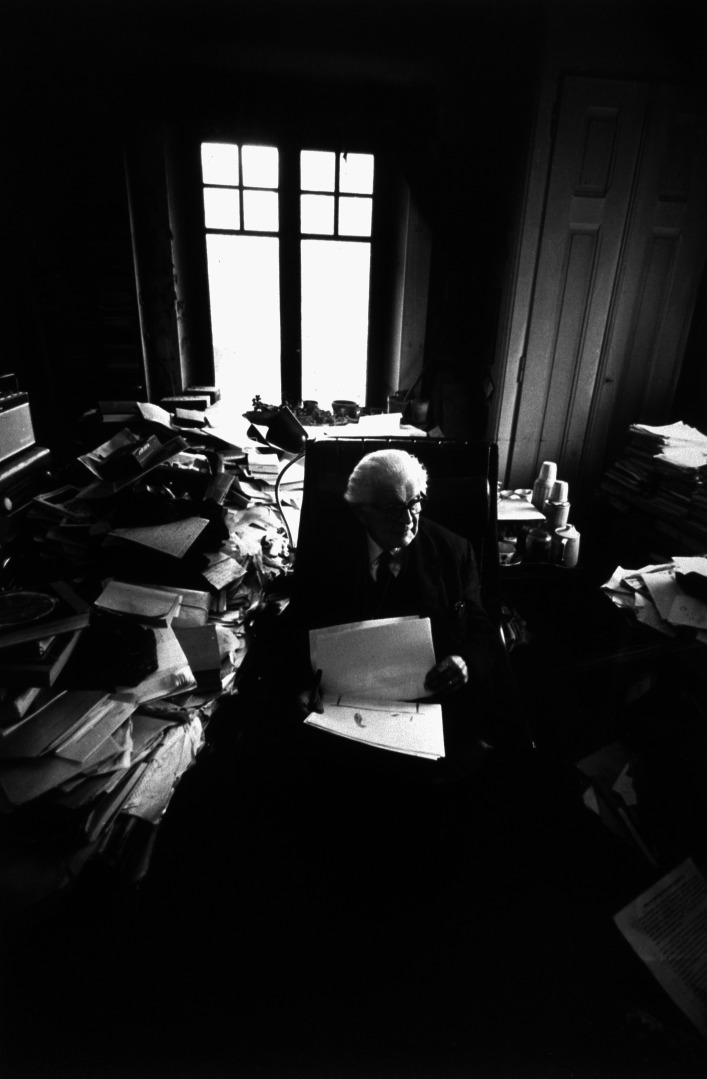
Photo of Piaget’s writing area (behind the seat). © Collection Photo Elysée, Fonds Jean Mohr, Photo Jean Mohr, 1978

**Fig. 8:**
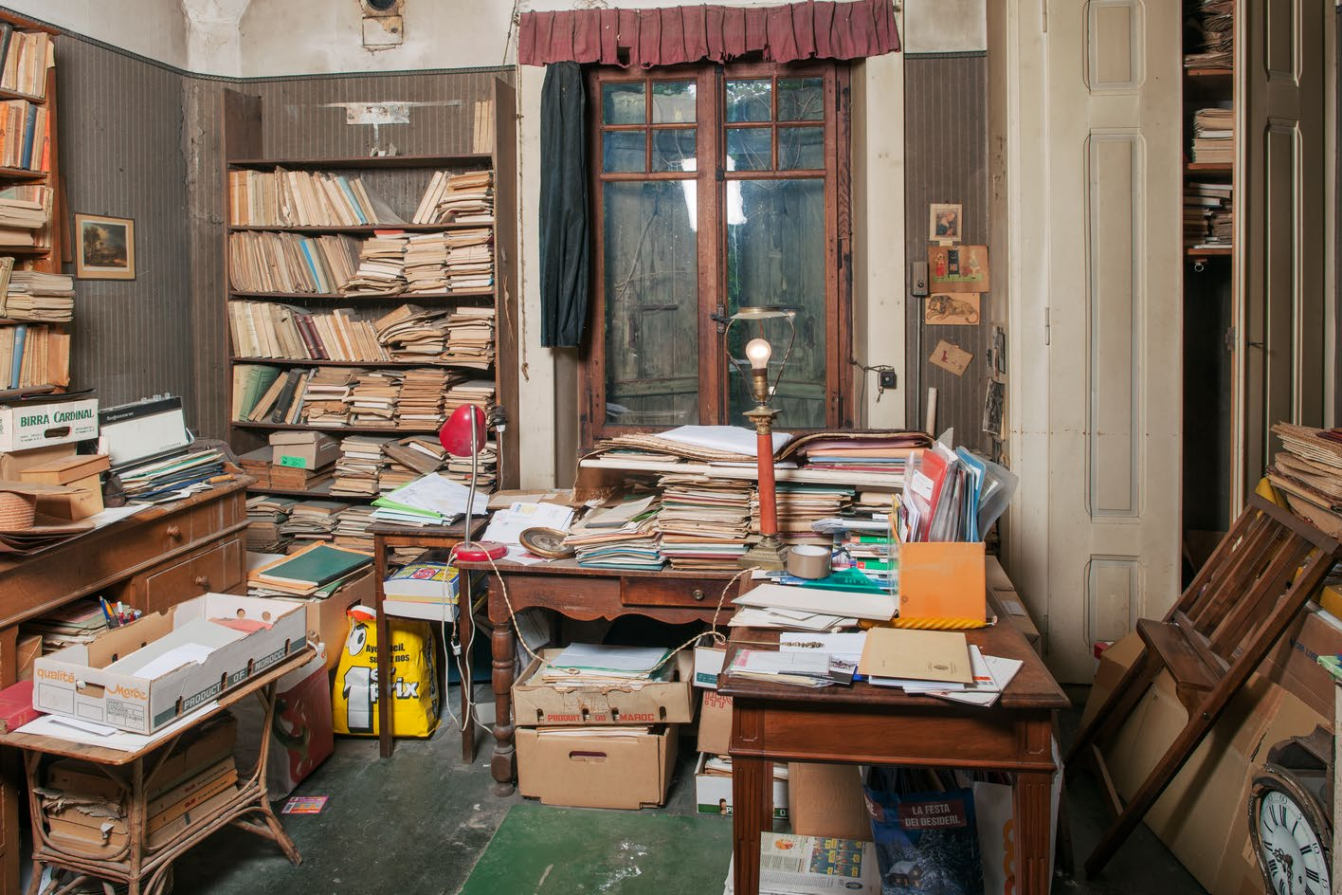
Same areas as Fig. 7 photographed in 2012. © Archives Jean Piaget, Photo R. Chappellu and D. Ponté

In this original photograph from the early 70s (Fig. 7), the areas left and behind Piaget were full of piles, boxes books, objects, etc. What became of these spaces when we entered the villa in 2012 is shown in Fig. 8: The picture was taken just from a place before Piaget’s armchair, and all the space that used to be taken up by stacks of books has been emptied. What’s more, a new element has appeared in the office stratigraphy: the primitive desk (on the center left) that was previously buried by the organization of the office.

**Fig. 9:**
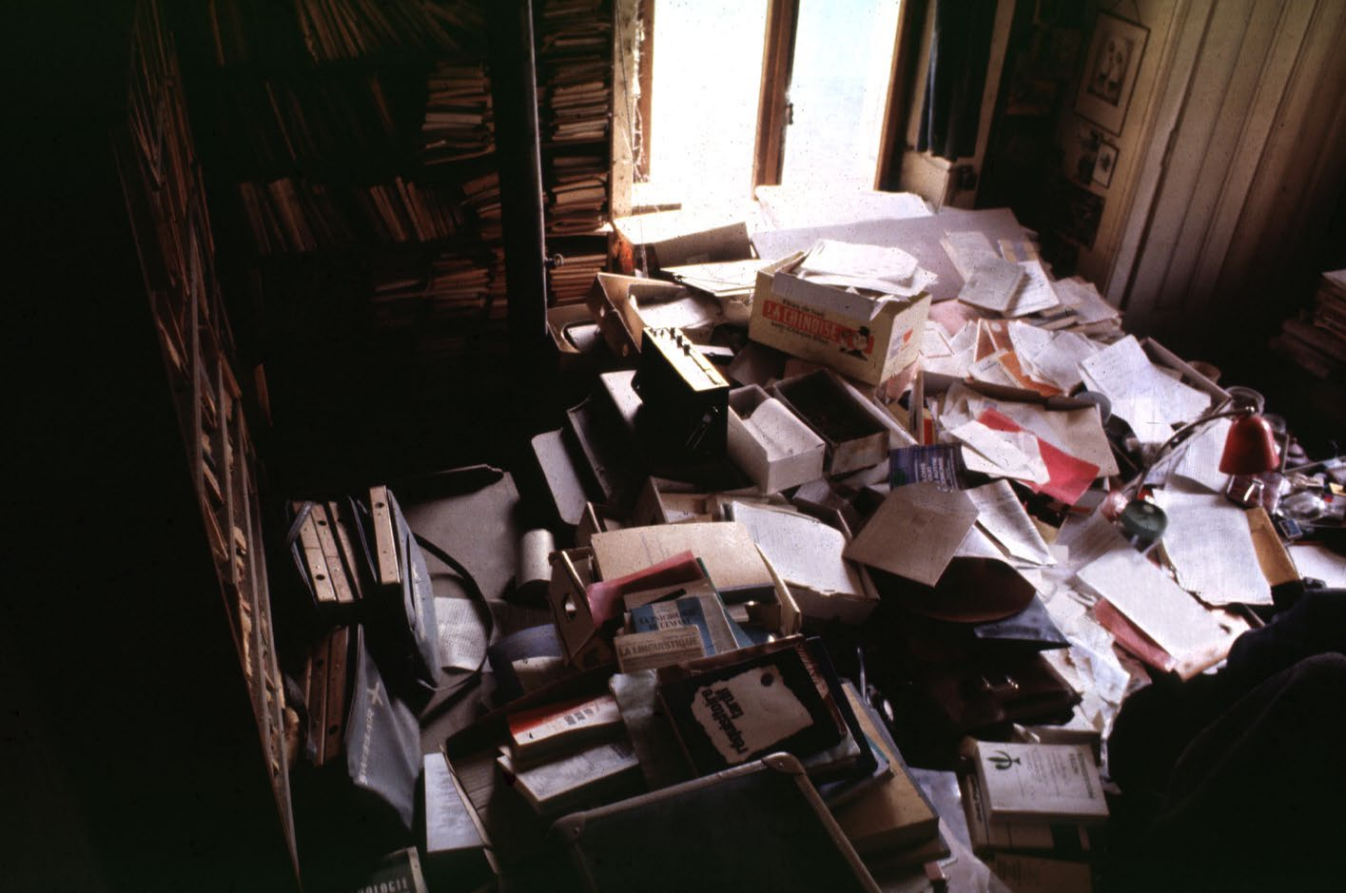
Overhanging photograph showing the south corner of the desk, center left. © Archives Jean Piaget, unknown photographer, c. 1978

Another photo, taken from a different angle, around 1978, provided greater precision and has allowed us to understand the fate of this primitive desk, parts of which (near the stove, whose pipe can be seen) were still visible (Fig. 9).

**Fig. 10:**
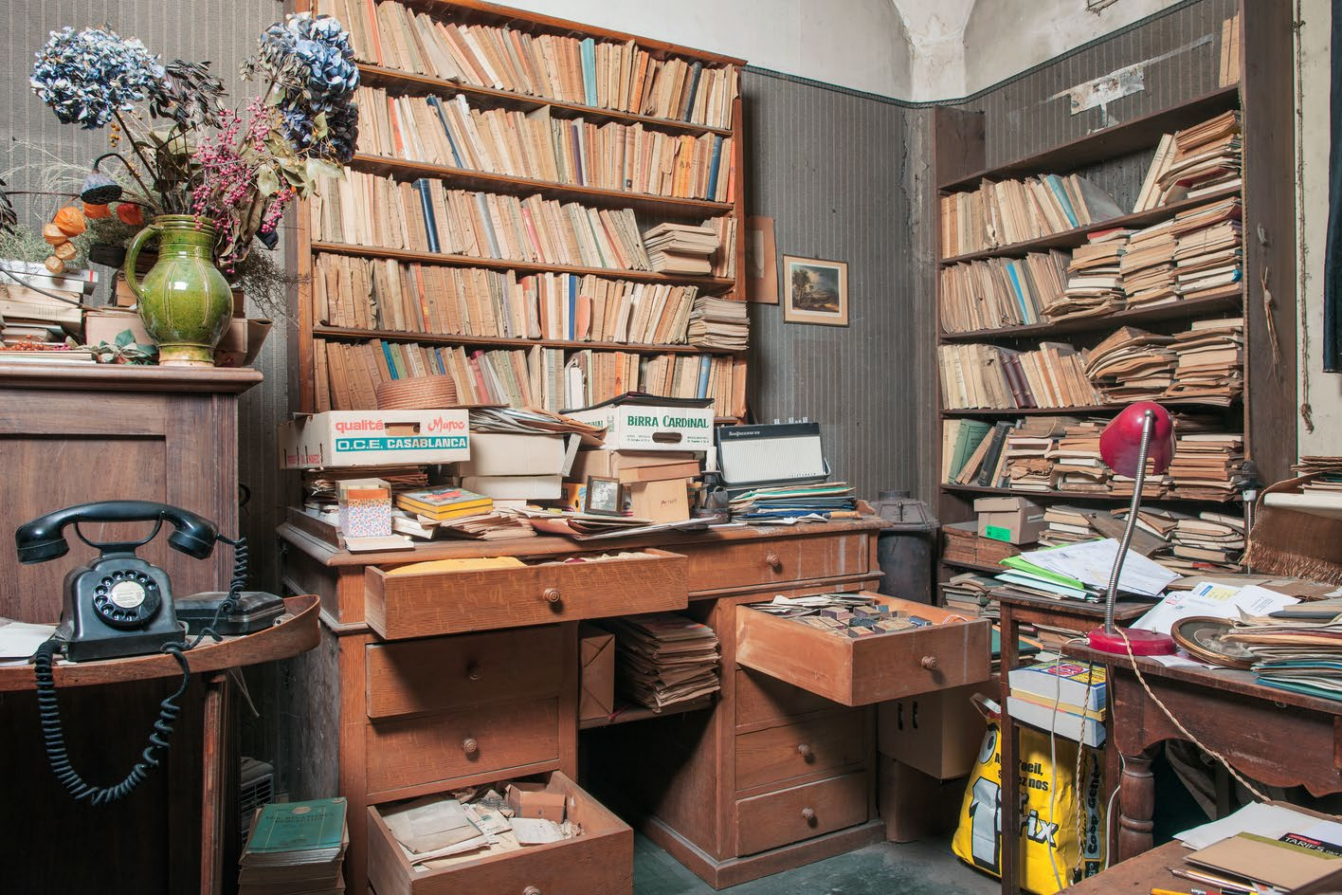
Primitive desk in 2012. © Archives Jean Piaget, Photo R. Chappellu and D. Ponté

By this time, the primitive desk had long since become inaccessible, blocked by several tables placed in front of it, perhaps with a board on top, forming the backbone of the archipilago in front of which Piaget’s writing space was located. This is why we can speak of interlocking desks, or in other words, of an initial desk that was placed against the wall, which was digested, assimilated by the now central desk that followed, developing from the periphery to the center in a kind of metaphor for the development of Piaget’s theory of internalization (Piaget, 1937: 311). A series of photographs from 1970, linked to Bringuier’s film, shows that the movement of the writing area went through several stages, which appear circular, like a mollusc: the primitive desk (Fig. 10) must have been in use since the Piaget family moved into the house.

**Fig. 11:**
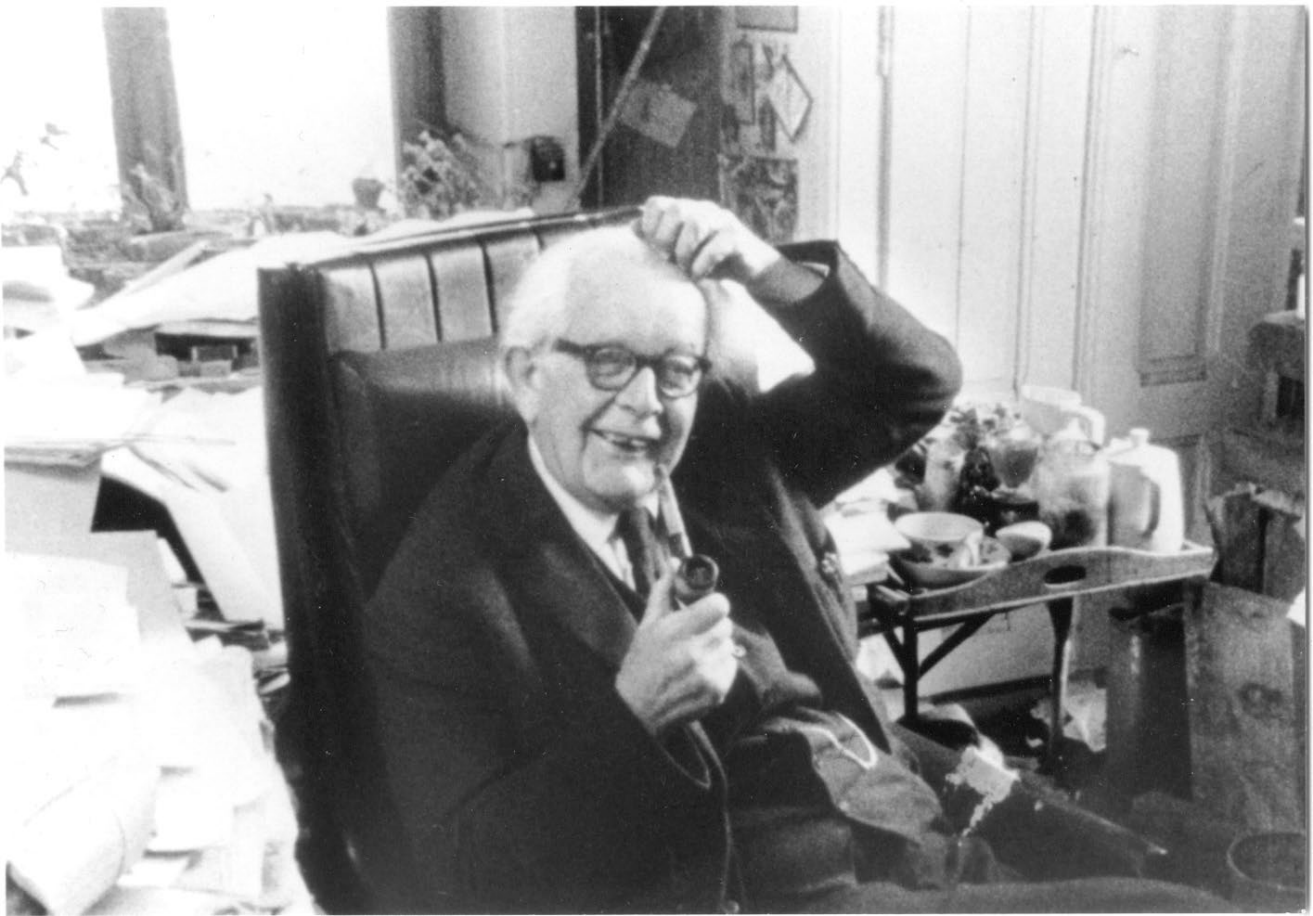
Second ‘desk’, 1969, behind Piaget. From the film “Un certain regard: Jean Piaget” by Jean-Claude Bringuier in 1969; alleged photographers Laszlo Ruszka and Michel Lioret

**Fig. 12:**
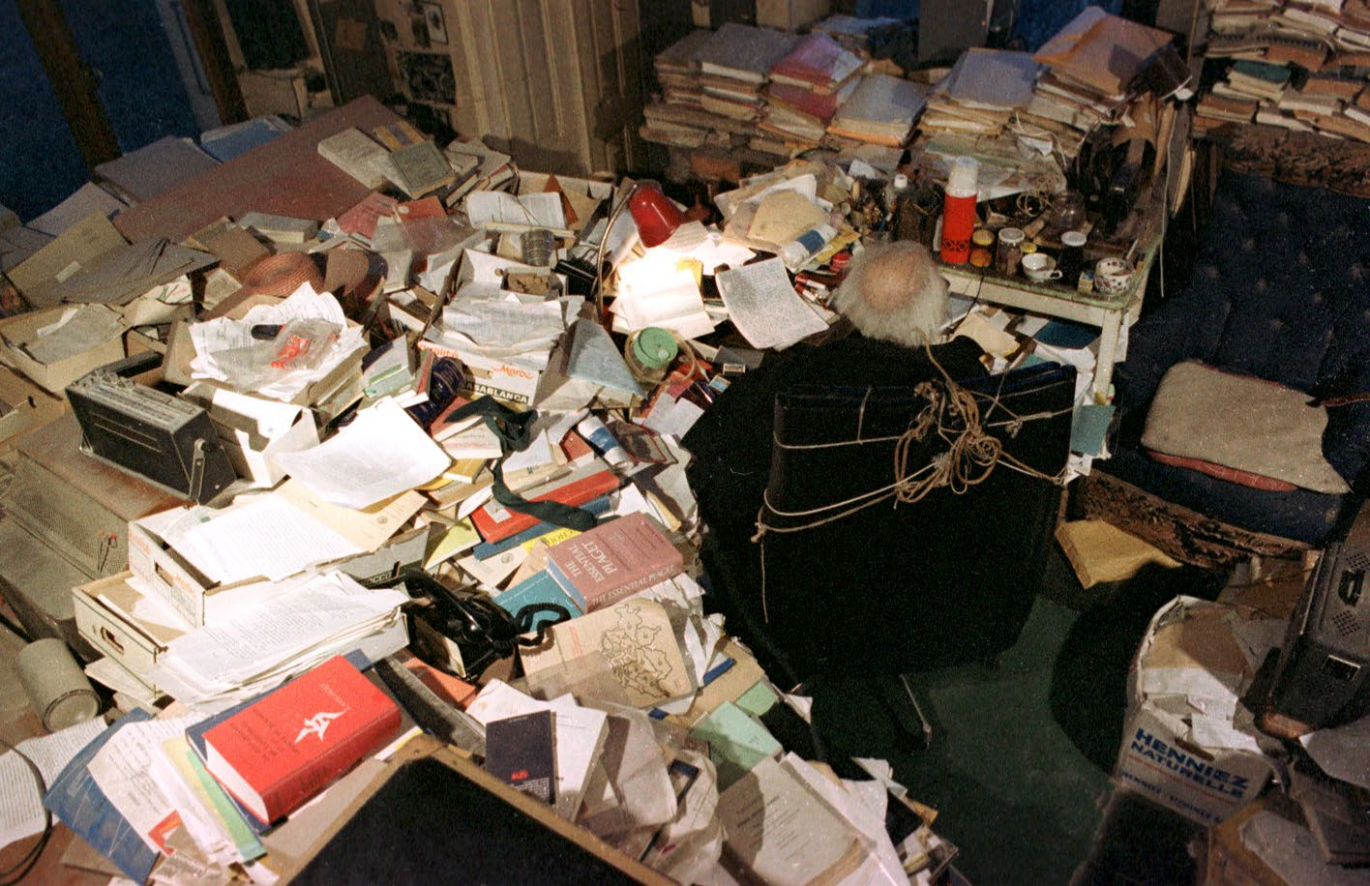
The Piaget workspace, circa 1979. © Archives Jean Piaget, unknown photographer

**Fig. 13:**
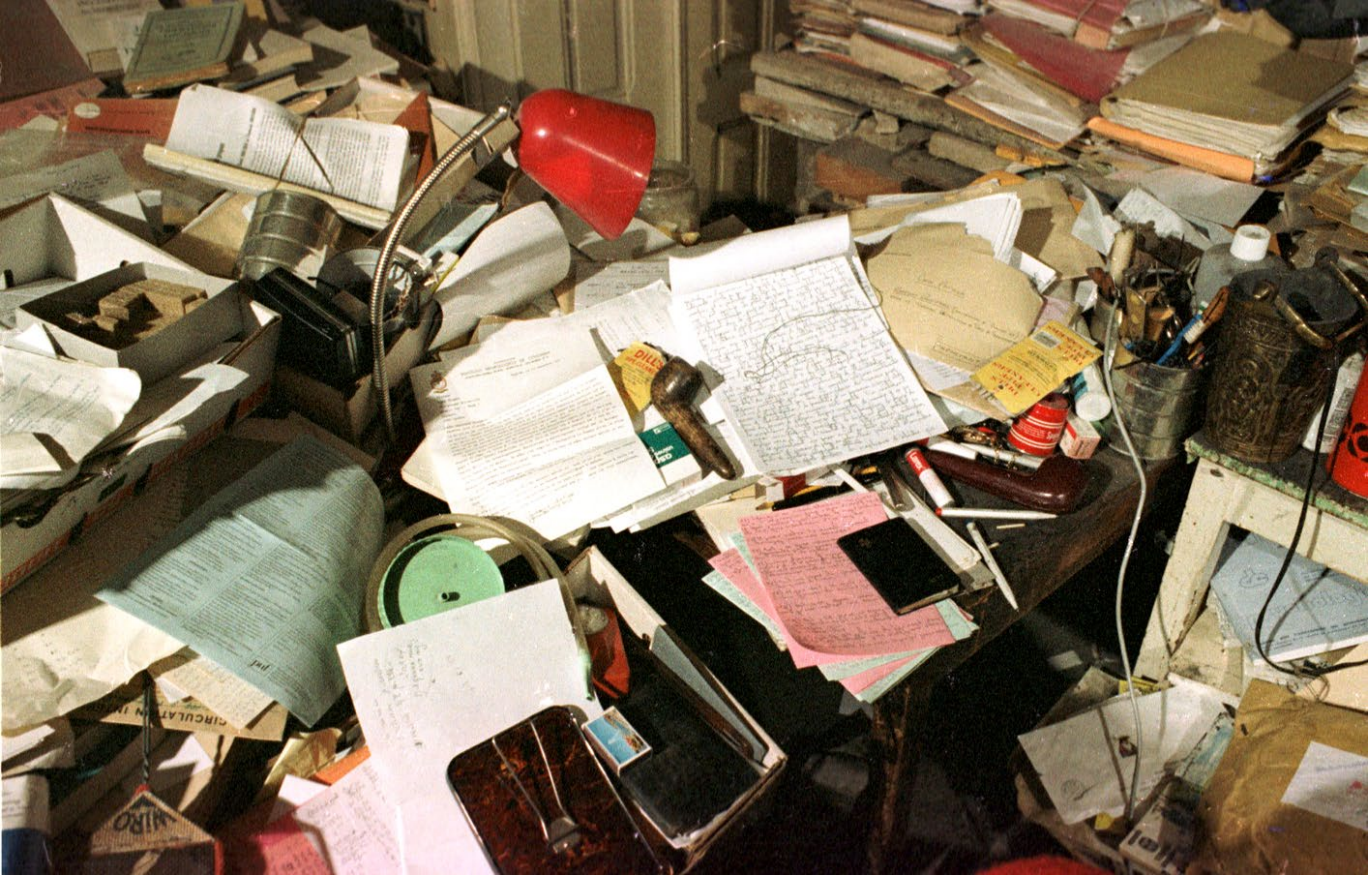
The passage to the cabinet (right of the picture) closed by the mobile writing area, circa 1979. © Archives Jean Piaget, unknown photographer

As can be seen in Fig. 11, this 1969 photo shows that the desk from this period was in the center of the room, leaving some space (in front of Piaget) for movement. The passage to the right cabinet (behind the cup tray) was still open. It allowed to reach both the stacks before the two windows as well as the plants *sedum* which Piaget placed in front of the south window, visible on the top left. Ten years later, the extension of the archipilago closed this passage and further reduced the circulation space, as shown in the 1979 photo (Fig. 12 and especially 13). The sedum disappeared in Fig. 12 and the passage is clearly closed in Fig. 13, showing the ‘third’ desk. Therefore the “desk” was mobile, a moving writing workspace that adapted to the volume of goods received and processed. If the office had been photographed around 1950 or 60, it would most likely have shown intermediate stages where the workspace was close to the desk then to the south window. All in all, we can visualize the initial stage (the primitive desk) and the final stages (from 1970 to 1979), but the intermediate stages of the mobile office within the archipilago probably took the form of a curve––initiating a spiral like the growth of a mollusk. This intellectual ecosystem was mobile and it seems that Piaget added a new table to the office each time it became necessary.

## The Second gift, the Piaget Family Donation of 2012

**Fig. 14:**
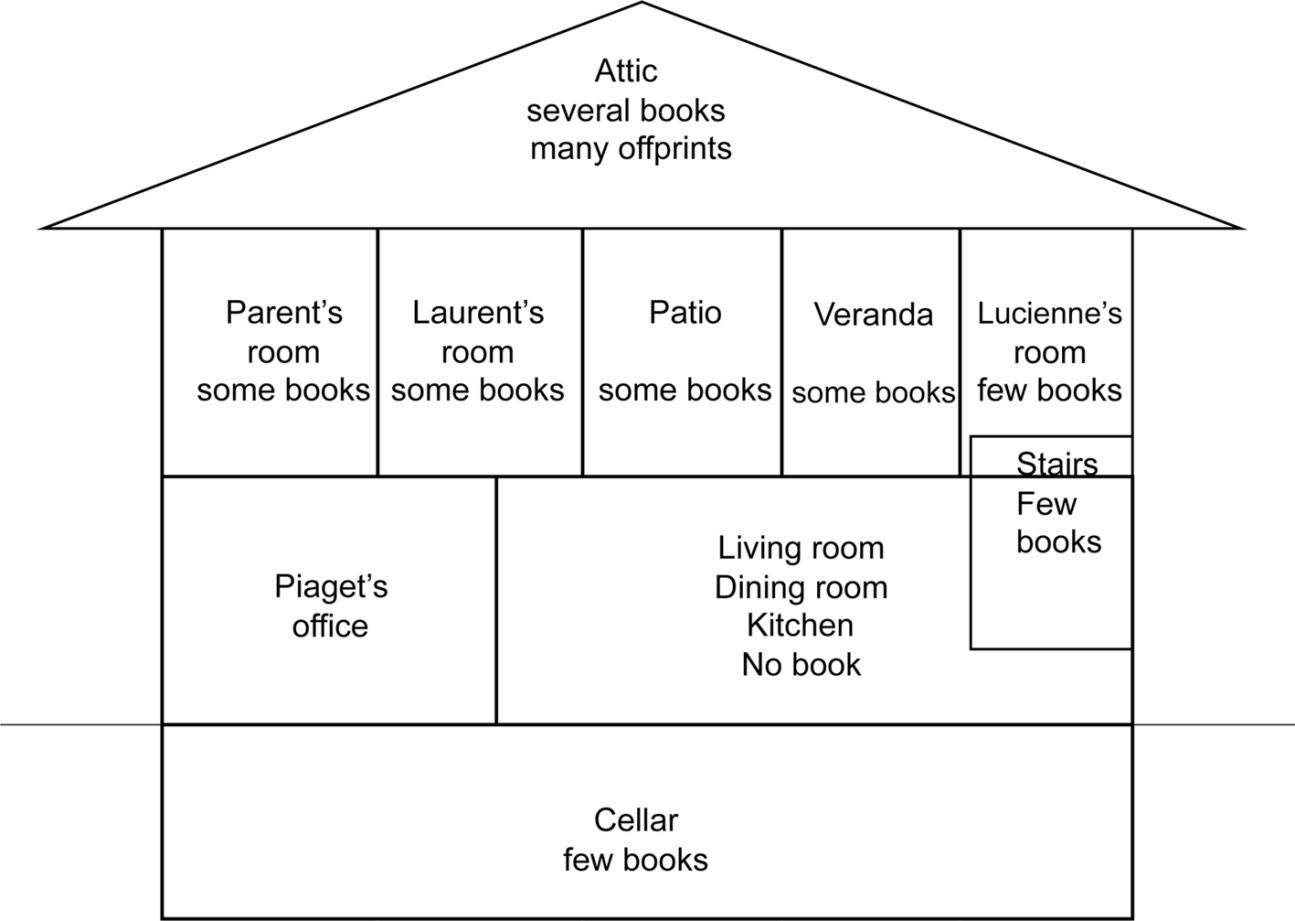
Distribution of Piaget’s books in the house *Les Cerisiers*

If the story of the first donation could be described as complicated, the Piaget family donation presented other challenges, much more so for the archives than for the library. The agreement signed in 2012 gave the Piaget Archives “all the intellectual content of the Villa”^1^. That’s about 80’000 archival documents, including the rest of the library; Yet we’re not just talking about Piaget’s library, but also the family libraries, especially those of his wife Valentine and, to a much lesser extent, his father Arthur. Before entering the villa, and especially before moving anything, the Piaget Archives decided to carry out a photographic campaign of Piaget’s office. The aim was to document the organization of the office as we found it. However, the agreement that entrusted the Archives with the intellectual content of the entire villa, which was no longer inhabited after Laurent’s death, created a new spatial problem. In other words, we were authorized to search the entire house for archives and printed matter in any place. And many places turned out to be unexpected reservoirs of books and archives. Examples include the series of bound PUF books given to Piaget in 1976, which were kept in the library in Laurent’s bedroom, or the piles of articles found in the attic, from the 1940s and 1960s, some of which were dedicated to Piaget. We had to choose criteria for attributing certain works to an owner, and fortunately a good part of Valentine’s books bore her signature. As for Piaget’s books, practically all the academic literature could be attributed to him. Although it was generally possible to identify the holders of libraries, their spatial boundaries were somehow blurred and it was often impossible to know how the book circulated between the various rooms. Piaget’s books were found in practically every room, as shown in Fig. 14.

To attribute a book to Piaget, especially those that were not located in the office, we used the following criteria: firstly, manuscript criteria, such as the presence of a signature, marks or notes by Piaget in the work, a dedication, a bookmark in Piaget’s handwriting or a letter stuffing a work; and secondly, thematic criteria, such as the work’s belonging to the disciplinary fields of psychology, philosophy, science, epistemology or education.

Let’s now go back to our issue related to the relationship between the researcher and the order.

## II. Understanding an Intellectual Ecosystem

### Order and Disorder

If every researcher is confronted with the question of disorder and order, the case of Piaget’s office is an extreme one that has become iconic thanks to Jean Mohr’s photographs and the accounts of some of his students and journalists in the 1970s. This is what makes it so interesting and perhaps emblematic of the relationship between the researcher and his worktools. In fact, the office is a symbolic object, famous for its disorder, which helped to situate Piaget in social representations of the scientist and psychologist as early as 1961, when one of Piaget’s students, Joan Bliss reported on it in the *London Time* (Noël, 2020: 273–274).

**Fig. 15:**
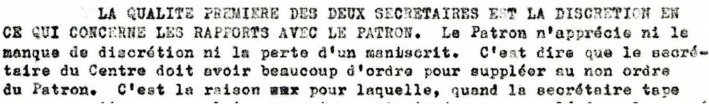
Excerpt from the center secretary’s “disorder from the boss” specifications

The images of the office became emblematic of the contrast between the mental order of a theory and the material disorder of an intellectual. Moreover, Piaget’s disorder was also famous at the school of Geneva, to the point of being integrated into the *International Center for Genetic Epistemology*’s work routines: disorder was clearly an explicit fact of life, accepted and even publicly claimed, as can be seen from the specifications for the Center’s secretaries^2^. These specifications showed Piaget’s endemic disorder not only in his private office, but also in the way he managed research data at the Center (Fig. 15).

“In other words, the Center secretary needs a lot of order to make up for the boss’s lack of order.” As we can see, disorder was the subject of connotations and also humor that contributed to the Piagetian ethos as well as to his image, if not publicly, at least in the Piagetian environment. His way of dealing with disorder was captured by him with a good word, calling it “vital disorder”, in other words making it a property of the living, which seems rather like a cop-out, giving him a ready-made answer to avoid intrusion into his private life. A kind of naturalization of disorder, just as his theory is a naturalization of psychology. And yet, behind the “vital disorder” we’re actually dealing with a spatially multicontrolled environment. In fact, according to Laurent Piaget^3^, nothing was worse than a pile of books falling into the office, creating the *real* disorder, where the researcher lost his bearings because part of the intellectual ecosystem had been disrupted. There’s a lesson here that goes beyond the anecdotal: the library can reveal things about an author’s relationship to his works, as well as the organization he or she adopts within them. Thus, from the point of view of an author’s working practices, the kind of order and disorder he or she adopts is fundamental, and to understand it we need to look at it from the point of view of the organization of materialities in a place of knowledge such as the office.

## The Order of the Disorder

The intellectual ecosystem, of which the office is the most tangible and well-documented manifestation, involved distinguishing apparent disorder from material-intellectual organization. In Piaget’s case, the gap between the two was very wide indeed. The case of the mobile office shows that it is not only a question of spatial limits, but also of temporal ones, and that it has to be thought in spatio-temporal terms, i.e. in motion. In this respect, as a researcher, Piaget followed a kind of “law” of current things, but in a differential way. He used *semantic* objects linked to current content (books, texts, minutes, correspondence), while *functional* objects were never updated: for example, he never used a typewriter, a photocopier or a fax, all of which were available in the 20th century. Only the telephone sat to the left of the desk. By dealing only with semantic objects as goals, the organization of this intellectual ecosystem obeyed the law of topicality: things that have to do with current events were employed, while others acquired a dormant status. A model, seeking to identify the totality of the intellectual ecosystem by identifying its relevant boundaries, allow to account for that.

## The RBAB Model: Research and Archiving Behavior

**Fig. 16:**
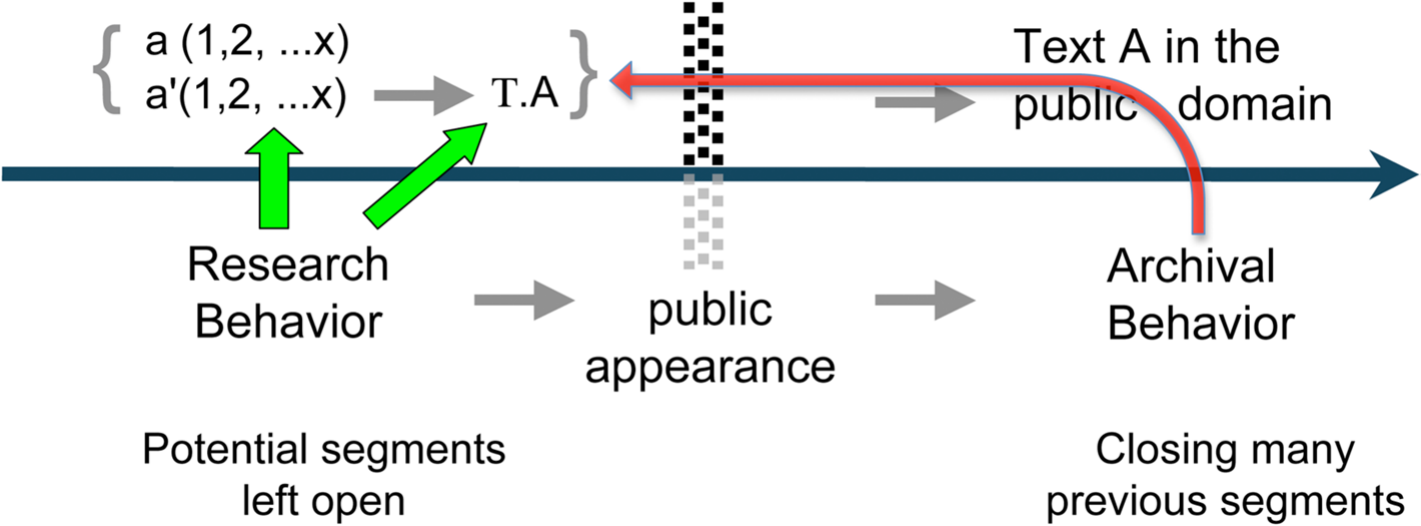
Transition from research behavior to archiving behavior (Ratcliff, 2015)

The RBAB model (Ratcliff, 2015) reflects on the fate of material archives in a world before the computer revolution. The specific aim was to understand the intellectual ecosystem of a researcher, using Piaget as an example. This model assumed that the core of the explanation for understanding changes in an intellectual ecosystem lied in the notion of *behavior*, understood in terms of the researcher’s *interactions* with segments of the intellectual ecosystem. In a research project, the researcher deals with series of segments––papers, books, articles, data, empirical surveys, material objects, notes, writings, notebooks, correspondences, etc.,––considered as a series which converges towards the drafting of a text *A*. At a given moment and according to a process academics are all familiar with, a result, in the form of a text, passes into the public domain, by means of a journal, a book, a communication, and today a website. This passage constitutes the main temporal boundary of an intellectual ecosystem, because it *generates semantic irreversibility*. In fact, in the intellectual ecosystem, it allows us to distinguish two main types of behavior: the first is the research behavior, which is characterized by leaving all the segments selected as useful for research in a potential, open state. However, when the text is made public, the researcher’s behavior changes and becomes archival (Fig. 16), always with respect to the same segments. As a manifestation of semantic irreversibility it is characterized by the closure of many previous segments.

The model, proposed in particular to understand a pre-computer environment, has shown that the main factor of significance is the public outcome of a research. This temporal boundary attributes a new role to the author, insofar as a scientific production, once made public, has a feedback effect on his or her behavior. The fact that it has been made public transforms the author into the *manager* of the publication. On the other hand, with regard to part of the intellectual ecosystem, the author adopts an archiving behavior rather than a research behavior: for example, he or she will no longer take care of certain research data, or certain archives related to this research. During the phase of research behavior––the counterpart of a research program that transforms the researcher’s cognitive skills––the segments used by the author (research data, notes, drafts, papers, books) remain *potential* and open in the sense of exploitable, realizable and reusable. Then, with archival behavior, however, some of these segments (research data, manuscripts, drafts, correspondence, even books) are closed. Obviously, RBs and ABs are often intertwined, especially when the researcher, like Piaget, works in parallel.

### Archiving Behavior Applied to Piaget’s Office

The difference between open and closed segments, based on both behaviors, was relevant to understanding the intellectual ecosystem represented by Piaget’s office. In this case, however, the segments changed scale: they were no longer objects, but groups of objects, and became areas of the office. In the photographs presented, we find several areas that were either inaccessible or untouched: The primitive desk and the bookcase near the stove (Fig. 9) became inaccessible at an unspecified time. The cabinet to the right of Fig. 7 which contained numerous research protocols from the 1920s and 1930s, and the bookcase next to the east window (Fig. 2, right window) were accessible but left untouched.

A second aspect of the RBAB model’s relevance to the office ecosystem deals with another temporal boundary: the author’s death. In 1980, Piaget’s death shifted the intellectual ecosystem towards a system archive, signaling the phenomenon of delegation of the behaviors. However, the disappearance of the author’s research behavior does not mean the disappearance of all research behavior, as shown by the publication of a number of works after his death (Piaget, 1981, 1983, Piaget & Garcia 1983, 1987). Archiving behavior was also delegated to other actors in the system. With Piaget’s death, his AB was delegated to his wife Valentine and his son Laurent, but only the latter seems to have taken this path, limiting his action to stacked works, according to a criterion of verticality or horizontality, without being exhaustive. Files were removed from the horizontal piles and the books were grouped together to form the first donation circa 1996. Consequently, there was a continuity in the types of behavior, though they led to different results. After Laurent’s death in 2011, the delegation was made to his two sisters, Jacqueline and Lucienne, who in turn delegated the archival tasks to the Piaget Archives. Therefore there are indeed temporal boundaries that have affected the space and the management of the disorder, modifying its meaning and the system of practices associated with it, as shown by the burial of the primitive desk by the archipilago.

In this framework, the time of the archivist’s, librarian’s, journalist’s or historian’s intervention is factored into the equation. Indeed, the history of authors’ libraries (Belin, Mayaux & Verdure-Mary, 2018) has often been written at the time T + 1, that is, *after* final intervention by the author, his or her heirs or professionals who came to reclassify and modify the original order. Rarely, some had the privilege of preserving the author’s order – such as Cudré-Mauroux (2018) with Starobinski’s library. Yet, the order observed is often a last order, which tells us little about the processes that made both the construction of the work and of the library interact. In the case of Piaget, as opposed to a single intervention at T + 1, we had the benefit of visual documentation of the changes that took place in the 1970s and later, and of two donations relating to different *areas*, thus possibly to a different time period. In other words, we found data on T-1, T, and T + 1. Visual reconstruction allowed us to capture certain folds in the history of the office-library, such as the mobile office. But what can we do, diachronically, to better reach the processes of interaction between the construction of the work and the movement in the office? To do this, we need new analyses that start from the heuristic edge of an intellectual ecosystem, moving away from visual history to work on statistical history.

### III. The Heuristic Perimeter of an Intellectual Ecosystem

#### The Heuristic Perimeter

**Fig. 17:**
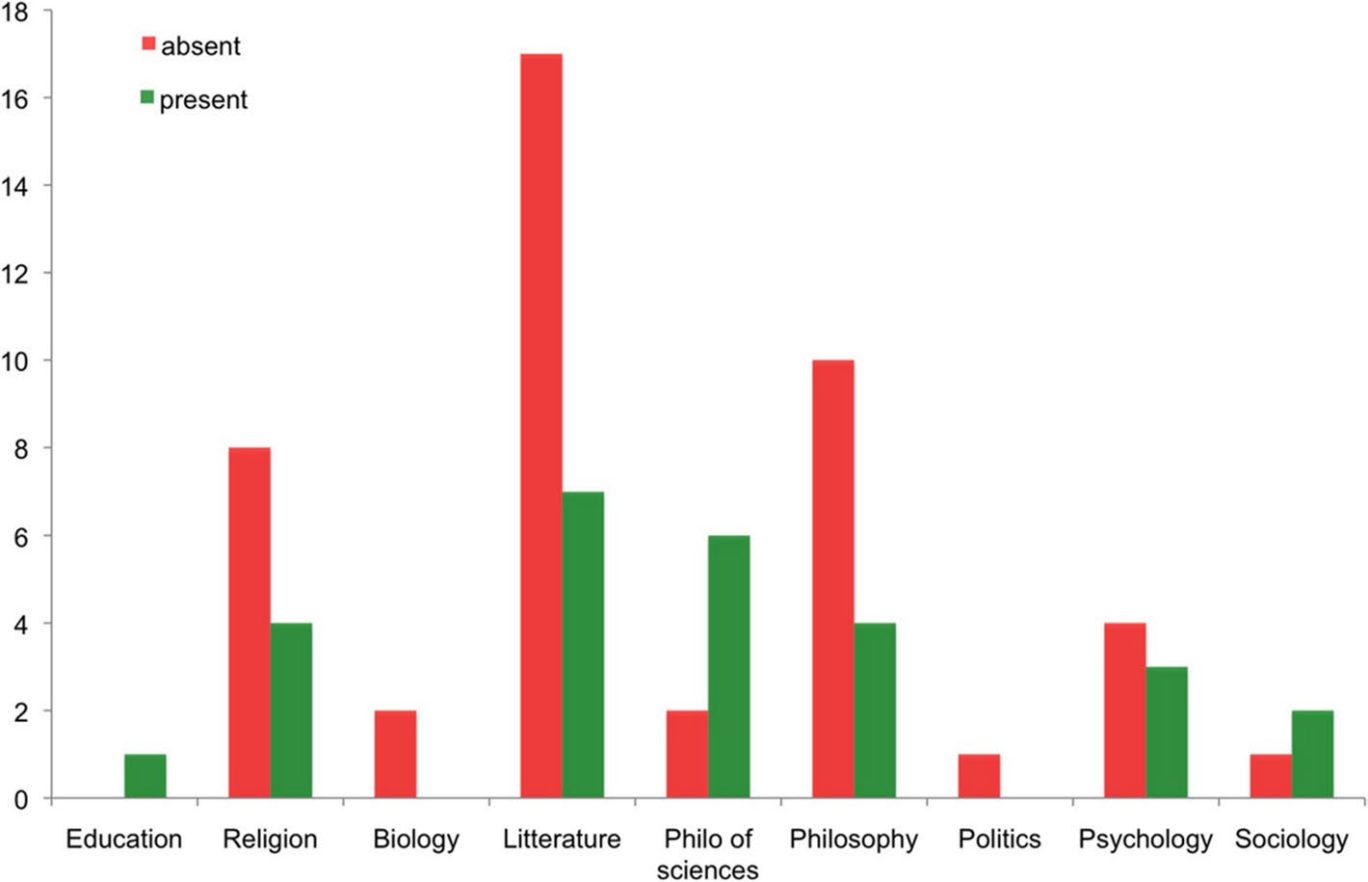
Thematic distribution of authors cited by Piaget in *Recherche* (1918), absent or present in his library

We call heuristic perimeter the set of results produced by the analyses of a library as part of its intellectual ecosystem. Many historians of library worked with such a heuristic perimeter. The analyses will provide a wealth of information on the genesis of Piaget’s works and give us an insight into Piaget’s relationship with the ideas and works of authors. They concern his reading practices, the mobility of his interests, his scholarly networks, his way of dealing with disorder, his relationship to interdisciplinarity, and the constitution of his library. In this respect, however, an intellectual ecosystem rarely keeps track of all its movements: purchases, readings, borrowings, and so on. Its owner does not record metadata, and access to metadata, especially as it relates to the genesis of the library, is contingent. For example, readings can be traced in the marks and annotations, but not the dates on which books were received. This leads to various limitations in the heuristic perimeter: some read and cited works were absent from the ecosystem, while other unread and uncited works are present. Figure 17 illustrates this by tracing the absence (45) and presence (27) in his library of works by authors Piaget cited in a 1918 text. Over 60% of the works Piaget red for this publication were missing from his library.

To understand how he built his library, we can invoke an age effect: in his youth, Piaget, who had few resources, used the family library and frequented public libraries in Neuchâtel and Paris (Liengme Bessire & Béguelin, 2008; Piaget, 1959). In the 1920s, however, he had more financial resources. This age effect overlaps with a reputation effect, which can be broken down into three factors: (1) as proactive aspect, Piaget adopted the practice of academic giving at a very early age, which contributed to the social construction of his reputation, as evidenced by a letter from 1921 in which he asked a journal editor for 70 copies of his first article for distribution^4^. This practice of academic giving was part of a culture of exchange among peers that Piaget continued with his books, which were always sent to numerous colleagues. In return, these colleagues sent him their works and publications, thus increasing his library; (2) the growth was also achieved through external requests for reviews and expert opinions on works, another classic practice in the academic world, as evidenced by the reviews Piaget carried out from 1921 onwards; (3) but the phenomenon that would play a much greater role, responding in part to the proactive aspect, was that of peer recognition, expressed in the sending of autographed works, which also played a role in increasing his library.

**Fig. 18:**
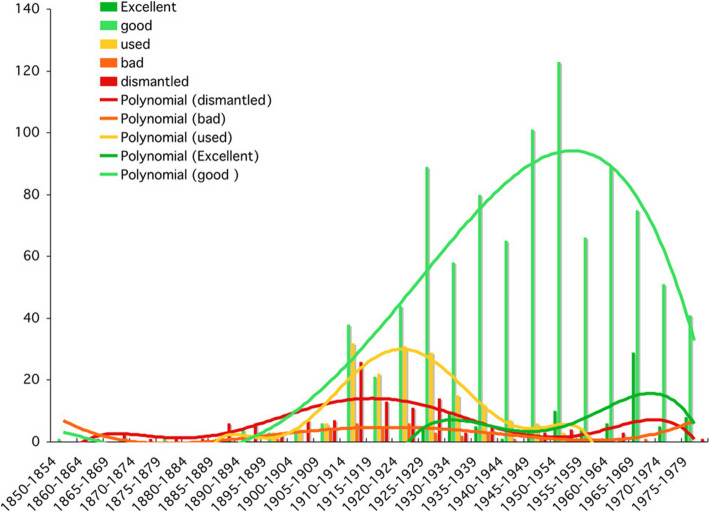
Frequency of books according to their condition per five years

**Fig. 19:**
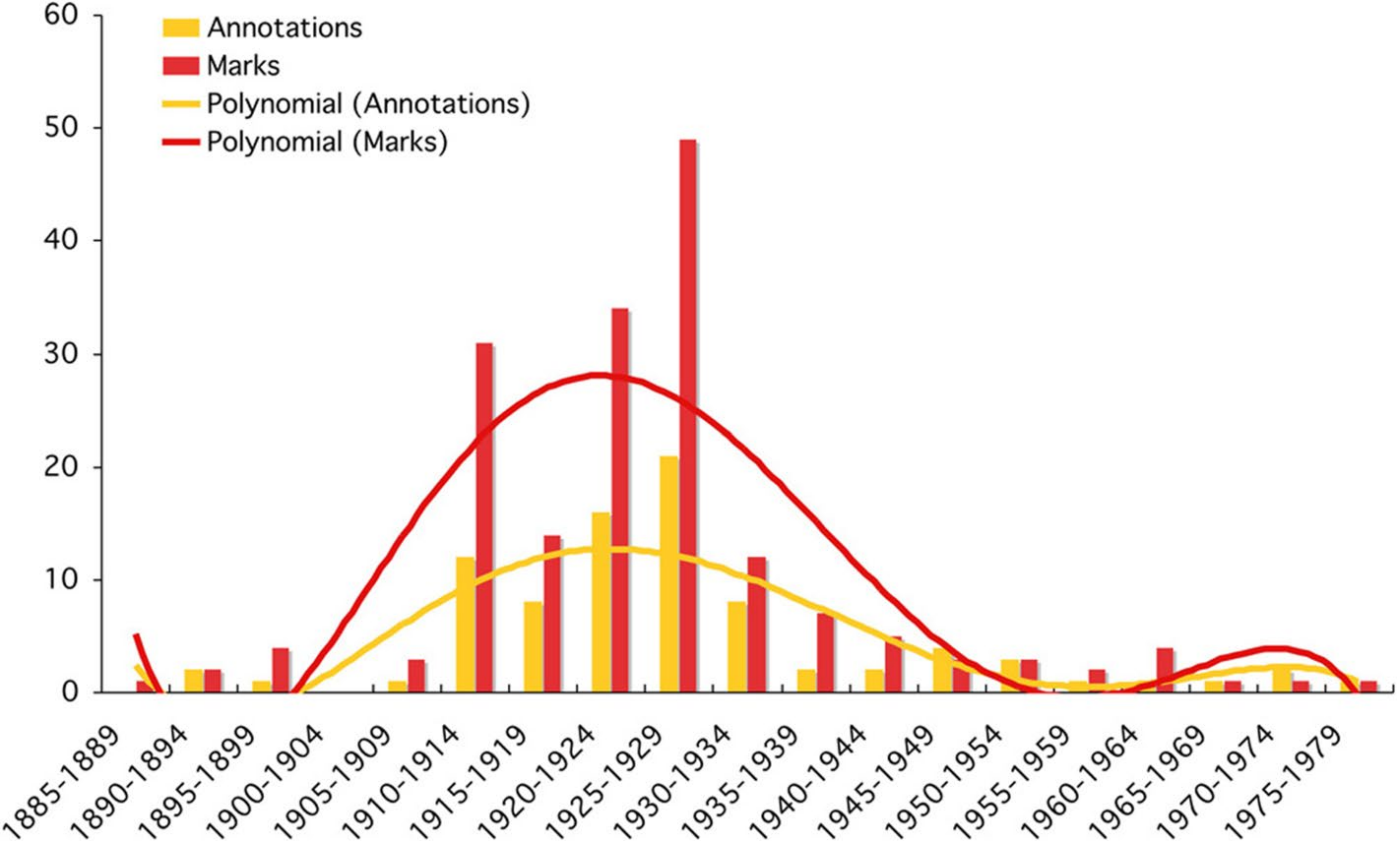
Frequency of annotated and marked books per five years

In a library, as has often been noted, books can be used to measure changes in the way people relate to reading. We can thus speak of the quality of reading, which can be measured by various parameters, including the condition of the book, annotations, and author marks. In the case of the Piaget Library (DFP), catalogued in a database currently being migrated to the University of Geneva, these fields (book condition, annotations, markings, dedications) have been filled in. This made it possible to highlight two periods in terms of book condition and reading intensity (Figs. 18 and 19).

Figure 18 showed that the books in the worst condition, and therefore the most widely read, dated from 1910 to 1939, while the books in the best condition dated from 1925 to 1975. This suggests a period of intense reading between 1910 and 1939, which is confirmed by the graph in Fig. 18 of the marks and annotations in the books, that showed a similar pattern. However, these data come exclusively from the DFP (second donation) and not from the BPP, whose corresponding fields were not filled in. These valuable clues point to a radical change in Piaget’s reading habits, from intensive reading (1910–1939) to a more diversified reading style after the Second World War.

**Fig. 20:**
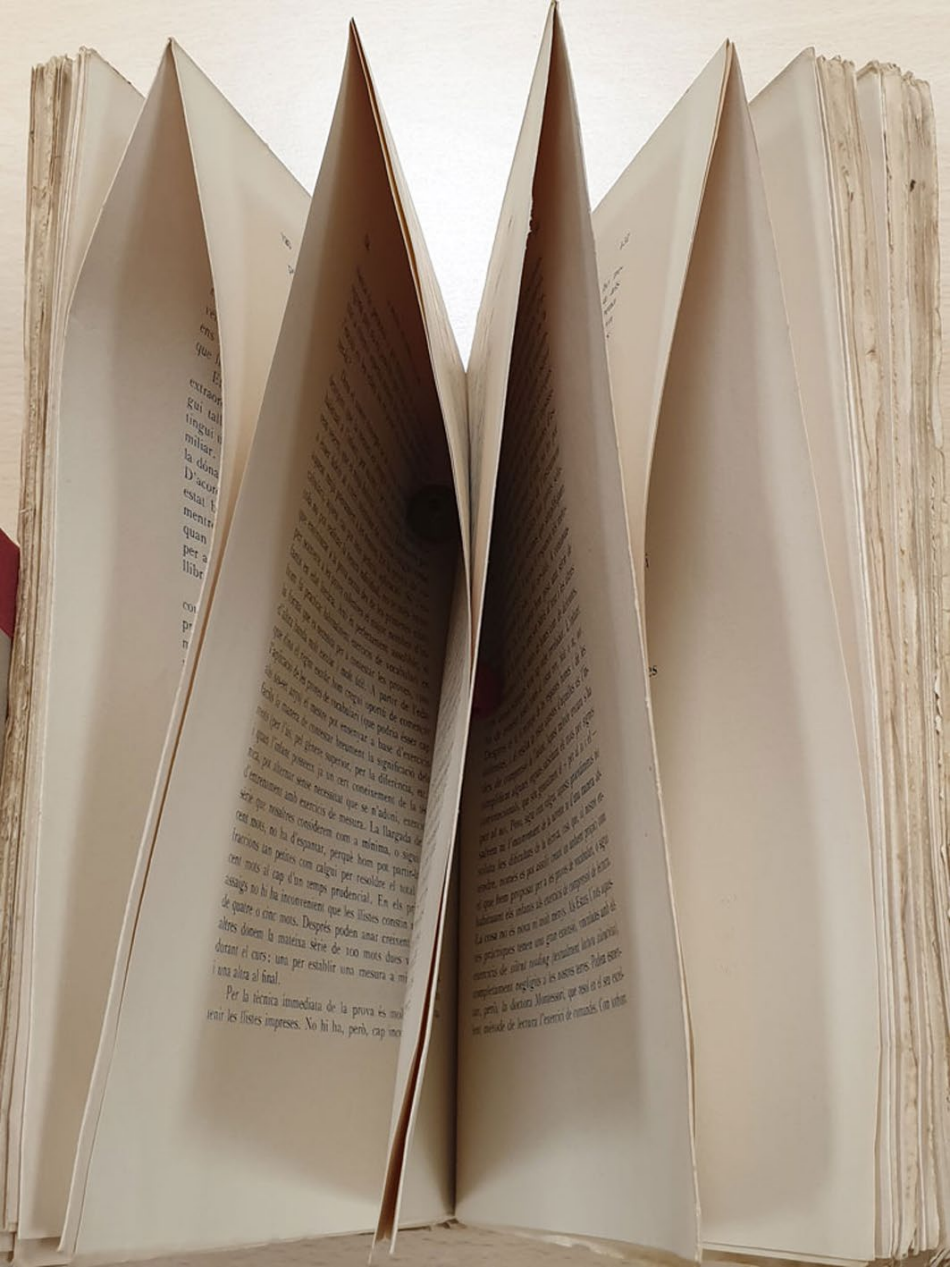
Uncut book, front view

In order to look more closely at these reading patterns, it is necessary to mention a peculiarity of the continental European book market, where a proportion of books were published *uncut* until the 1960s (Fig. 20).

An uncut book is the result of a printing process in which sheets printed in large A2 or A1 formats were *folded* and stapled, but *not trimmed*, by printers/publishers, who left this task to bookbinders or buyers. Part of the Piaget Library was made up of untrimmed books, whose characteristic feature is that they *cannot be read* unless they are trimmed. This is another valuable clue to the reading methods used by Piaget, a clue that does not exist in the Anglo-Saxon world, where even in the 19th century, books were cut by printers and bookbinders, but not by buyers.

But that’s not all. The *way in which the book was cut* (partially or completely) provided further information, since it can be reinterpreted in terms of open and closed segments, and a new type of book-related behavior can be identified: (1) In the case of uncut books, the segments remained closed, which is an expression of archiving behavior; (2) fully cut books show that at some point they have become open segments, which led to intensive reading; (3) Partially cut books, on the other hand, allowed partial reading, which was sufficient for the reader to grasp the usefulness of the work. But above all, these partially cut books are an important indicator of a new type of behavior, the *Control Behavior* (CB). This was based on *focal reading*, taking into account that many books of the 20th century contained author indexes that made it possible to determine whether an author was cited in the work or not. That’s what happens with an author-focused search engine, and this focal reading allows you to quickly find out what an author is saying in relation to your ideas. CB is therefore about two things: checking one’s scientific self-image as reflected in the literature and, more specifically in relation to Piaget, checking that one’s ideas have been understood and not hijacked by one’s readers, which was increasingly proving to be a challenge for Piaget. Finally, the CB helped to stimulate debate, as the scholar kept a watchful eye on the literature and what was being said about his theory, giving rise to controversy (for Piaget, see Parrat, 1993; Da Silva, 2023).

As we can see, in addition to the classical signs provided by annotations and marks attesting to the quality of reading, the cutting of a book, however material and non-semantic, becomes a trace of reading. In fact, a book cut down just to the author’s index and the pages mentioning him or her clearly indicates a focused reading.

**Fig. 21:**
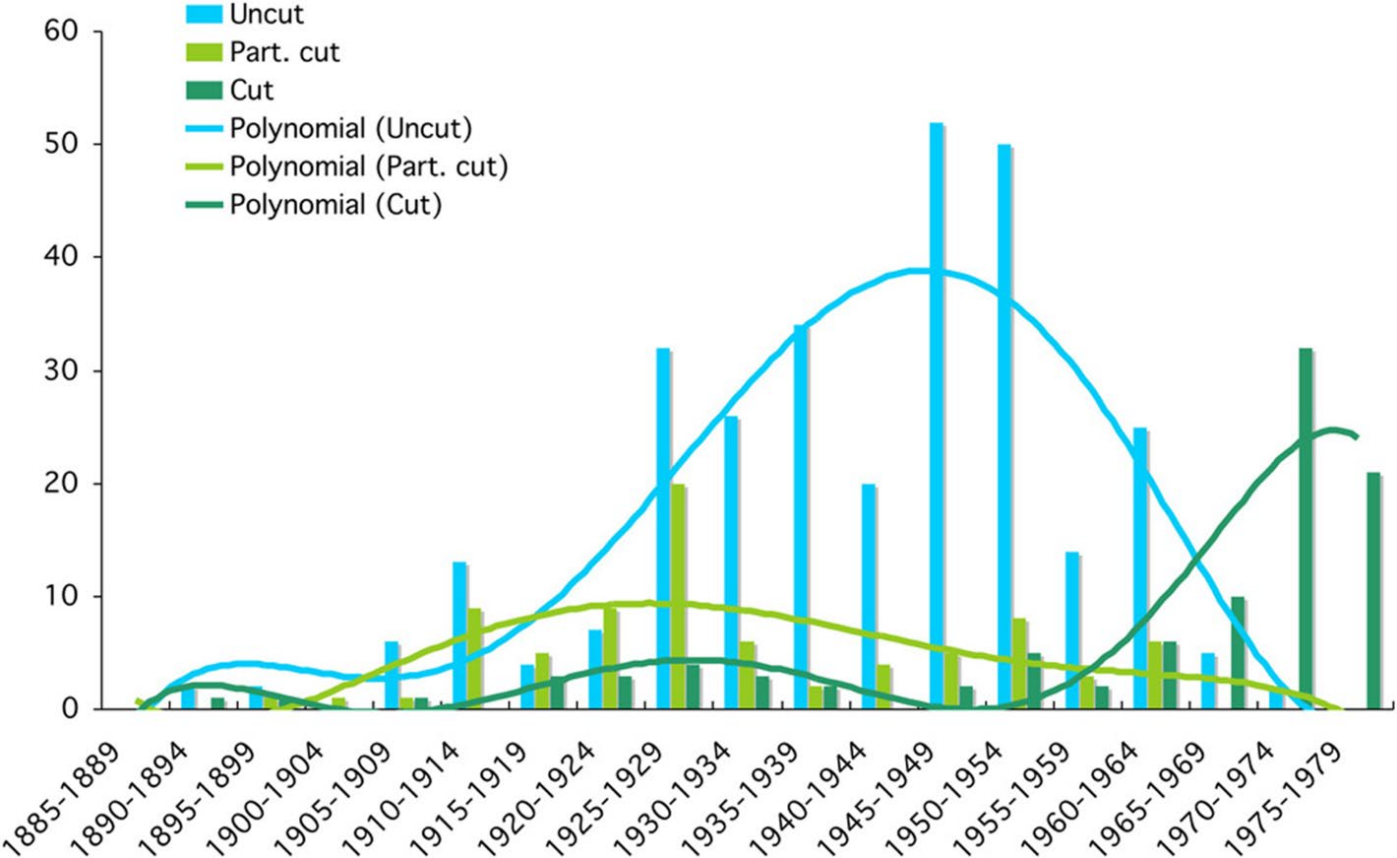
Frequency of cut, partially cut and uncut books in the DFP

The following graph (Fig. 21) shows the comparative frequencies of cut, uncut and partially cut items. It represents 24% of the items in the DFP library. Of these, uncut books made up a significant portion of the total. Circa 15% of the books in the library (DFP) remained uncut and dated mainly from the years 1925 to 1965.

### The Signed Library. The Geographical Distribution

**Fig. 22:**
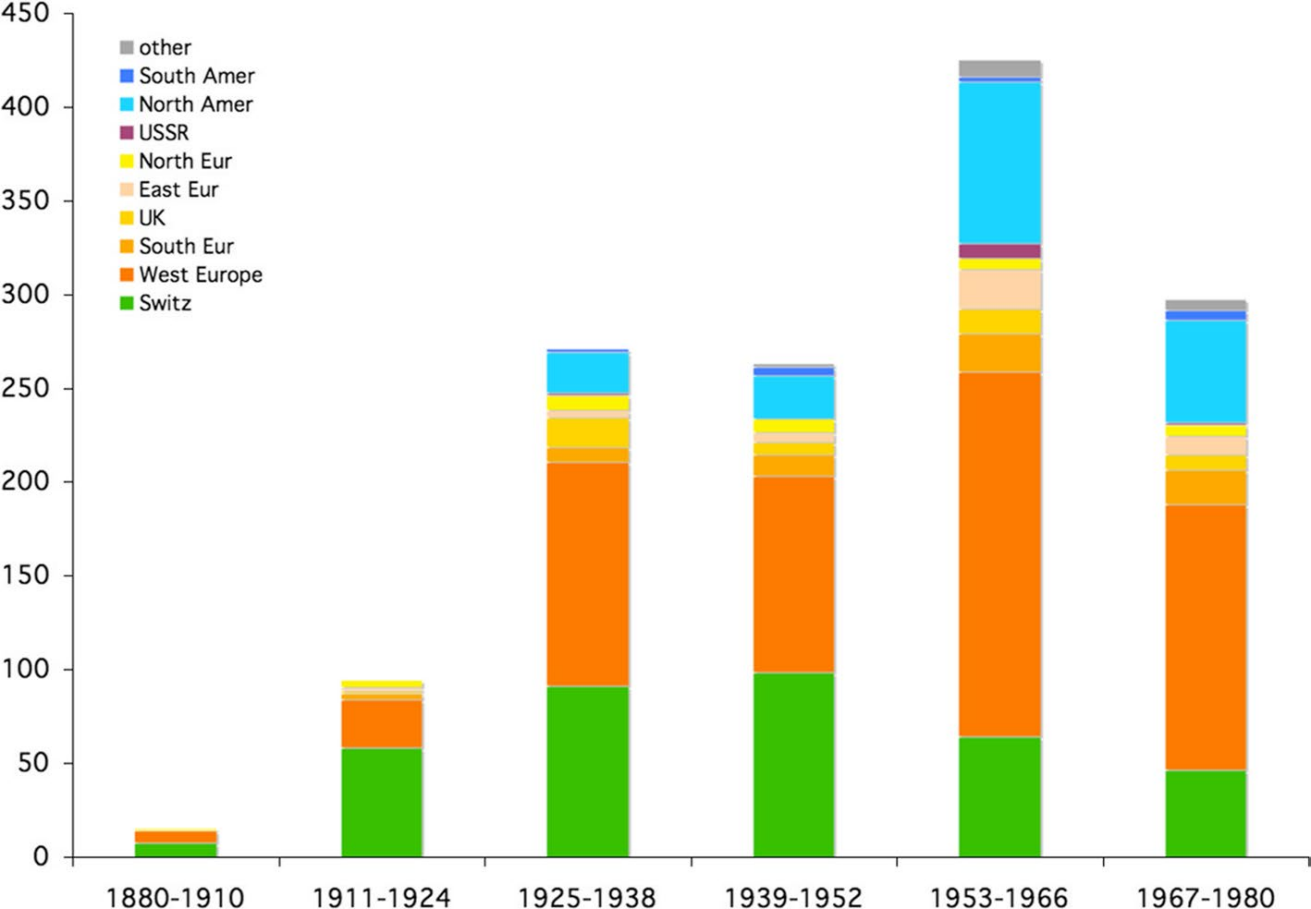
Frequency of signed books and offprints per continent over 14 years

The library can also tell us a lot about the author’s networks and reputation. In fact, in the pre-computer era, an author usually received offprints and books signed by colleagues. Gifts and exchanges became an integral part of the circulation of scholarly work. They can be seen as a sign of academic recognition, and in Piaget’s case, the signed part of the library was enormous, with at least 1353 signed texts, including 745 books and 608 offprints. Nevertheless, we can use the dedicated library to illustrate two aspects: the geographical distribution of the signed books on the one hand, and its interdisciplinary distribution on the other. The geographical distribution was analyzed by continent (Fig. 22).

It’s important to remember, of course, that the date of publication doesn’t always correspond to the date of signature, and this is especially true for Piaget’s early period and youth. Nevertheless, certain trends are clearly visible: Switzerland in green, Europe from orange to yellow, and the American continents in blue. This plot shows that after the first scientific recognition by Swiss academics and some Europeans very early (at the age of 14–15), the international recognition began around 1925 and continued until after the Second World War, with Europe and then Switzerland dominating. Worldwide recognition began in the 1950s, when the United States (and Canada) took second place after Europe, while USSR, Japan and Australia, as well as a number of African and Asian countries, became part of the picture. In the North America case the international recognition can be correlated with the translations of Piaget’s work, especially from French into English, and with the perception of his *New Theory* (Burman, 2016).

### An Interdisciplinary Library

**Fig. 23:**
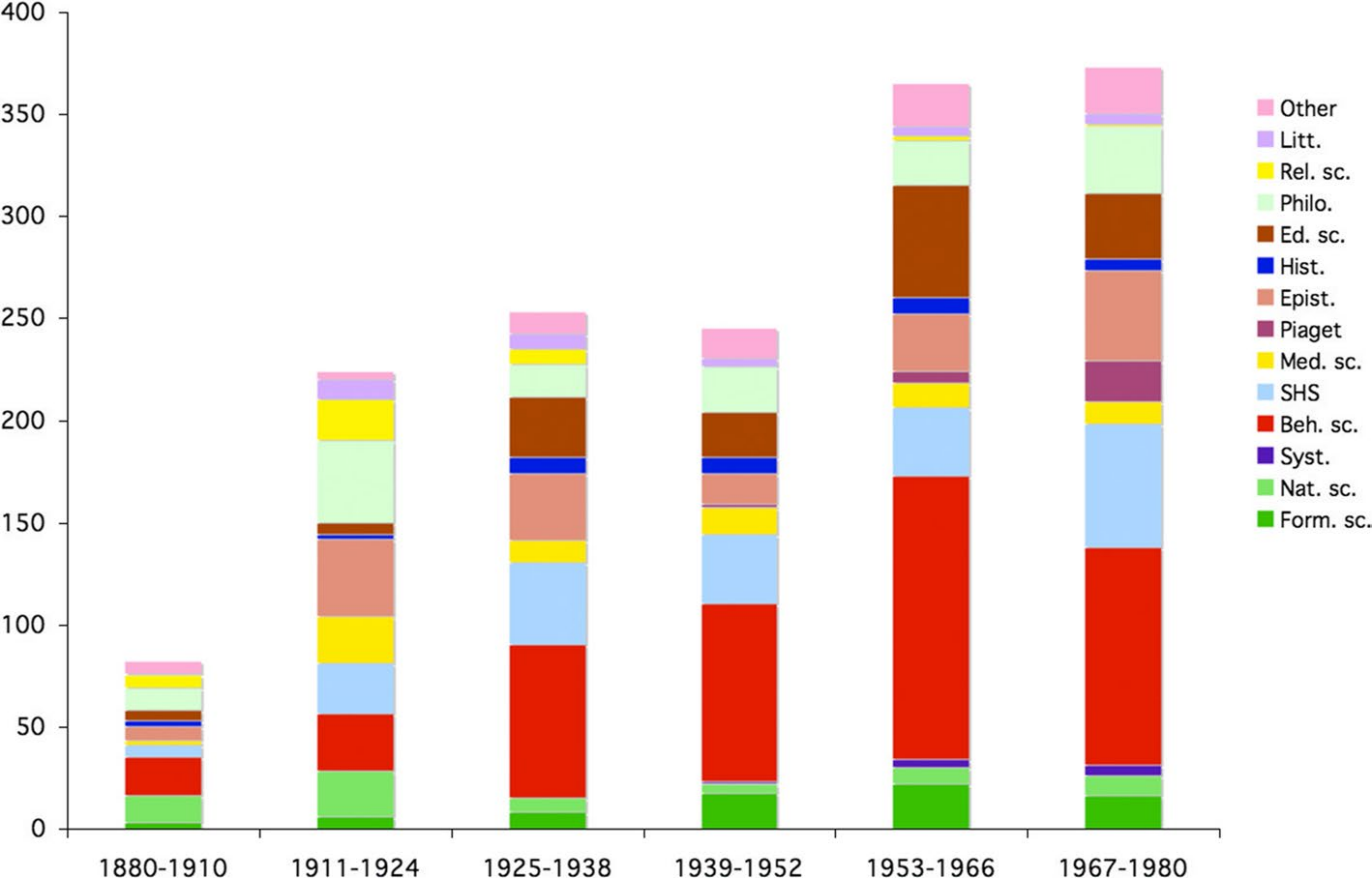
Piaget’s library without the signed items, frequency per period

Somes studies have highlighted the importance of interdisciplinarity in Piaget’s work (Chapman, 1988; Smith, 2009; Ratcliff & Burman, 2017). But, is interdisciplinarity reflected in Piaget’s library? To answer this question, we analyzed the range of disciplines in two sets of data: unsigned books and signed books. The first set was made up of works that, for the most part, were selected by Piaget and represented his research interests. The second set was complementary and illustrated the perception of Piaget by the actors of the scientific community. For the first set, I’ve added up all the books (DFP and BPP), from which all the signed texts were subtracted (Fig. 23). In fact, the autographed books were given to Piaget and not selected by him.

**Fig. 24:**
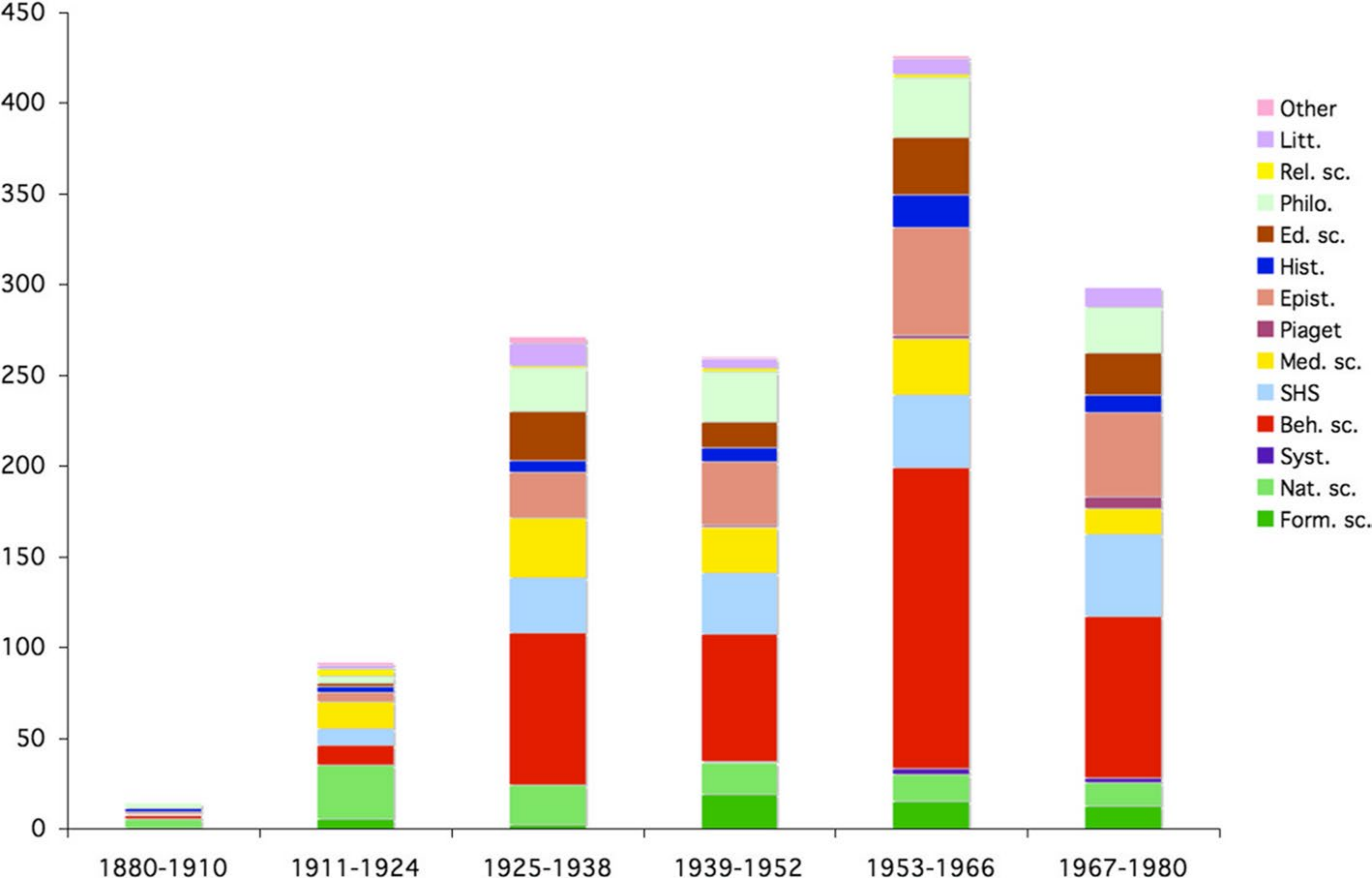
Frequency of signed books and offprints, per period

**Fig. 25:**
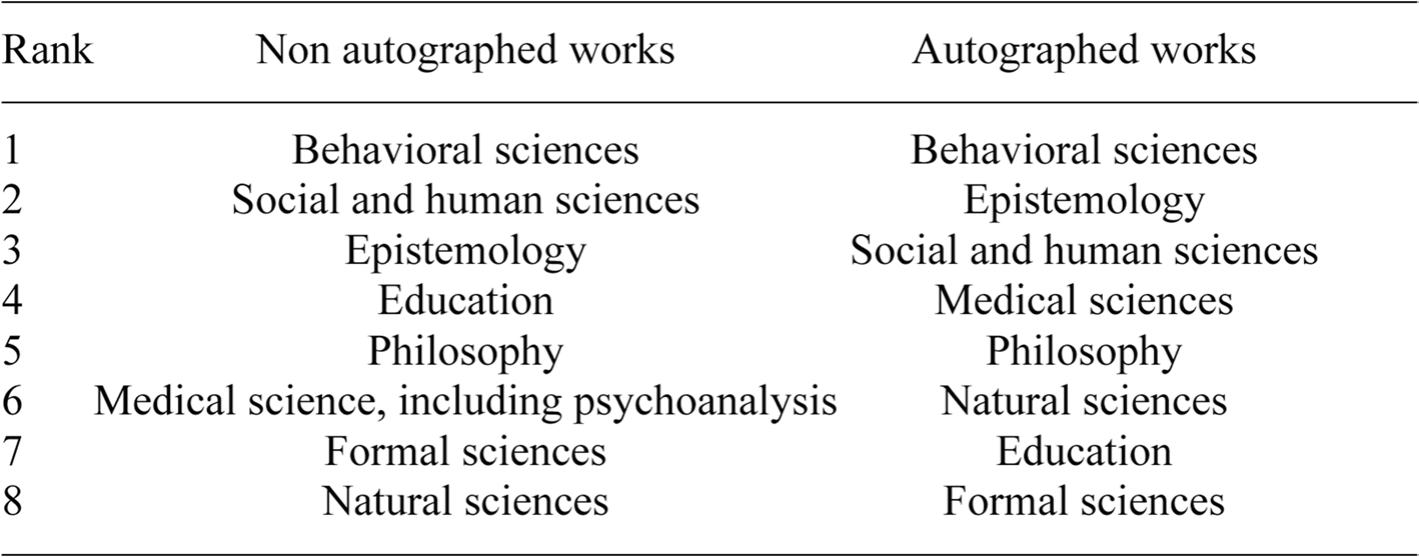
Ranking of the disciplines in signed and non signed books

The resulting graph (Fig. 23) showed a distribution across all disciplines in the natural sciences and humanities, with a predominance of the behavioral sciences, which included almost all of psychology. A distribution by rank showed the priorities (Fig. 25, left column). For the second set, the autographed library was subjected to the same analysis. The result was similar in terms of the range of disciplines, but the distribution by period was different. And the ranking followed a different distribution (Fig. 24and 25).

The data of figure 25 are difficult to interpret and show an interesting gap between Piaget’s own interests and the perception academics had of him. He was perceived more as an epistemologist and less as a social scientist, which is fair. And he was also perceived as concerned with medical disciplines more than by natural sciences or education, which was a surprise. This means that more actors of the medical disciplines than of natural sciences and education sent him their signed works. However, to come back to the interdisciplinarity issue, it was strongly reflected by Piaget’s library, both through his personal interests and in the perception of those involved in the most diverse scientific communities.

### Why the Archipelago? Comparison of the Two Donations and Libraries

Eventually, we shall discuss the ‘Arcipilago’ issue. As for why Piaget adopted this format for the office at a certain point, it was both a qualitative and quantitative response to both a change in program and to the increasing number of books and letters received, while the shelves were not only full, but becoming inaccessible. Historians have been sensitive to this, and for Jacob (2001: 53), for example, “accumulation produces significance effects”. In Piaget’s case, ordering, either due to accumulation or usage, was a waste of time. He believed, reported Bringuier, that “organising your workspace in this way takes far less time than tidying it up every day” (Noël, 2020: 275). Therefore the time that others devoted to vertically arranging books, librarianizing their works by arranging them in a certain order of their own, he devoted to researching and writing.

**Fig. 26:**
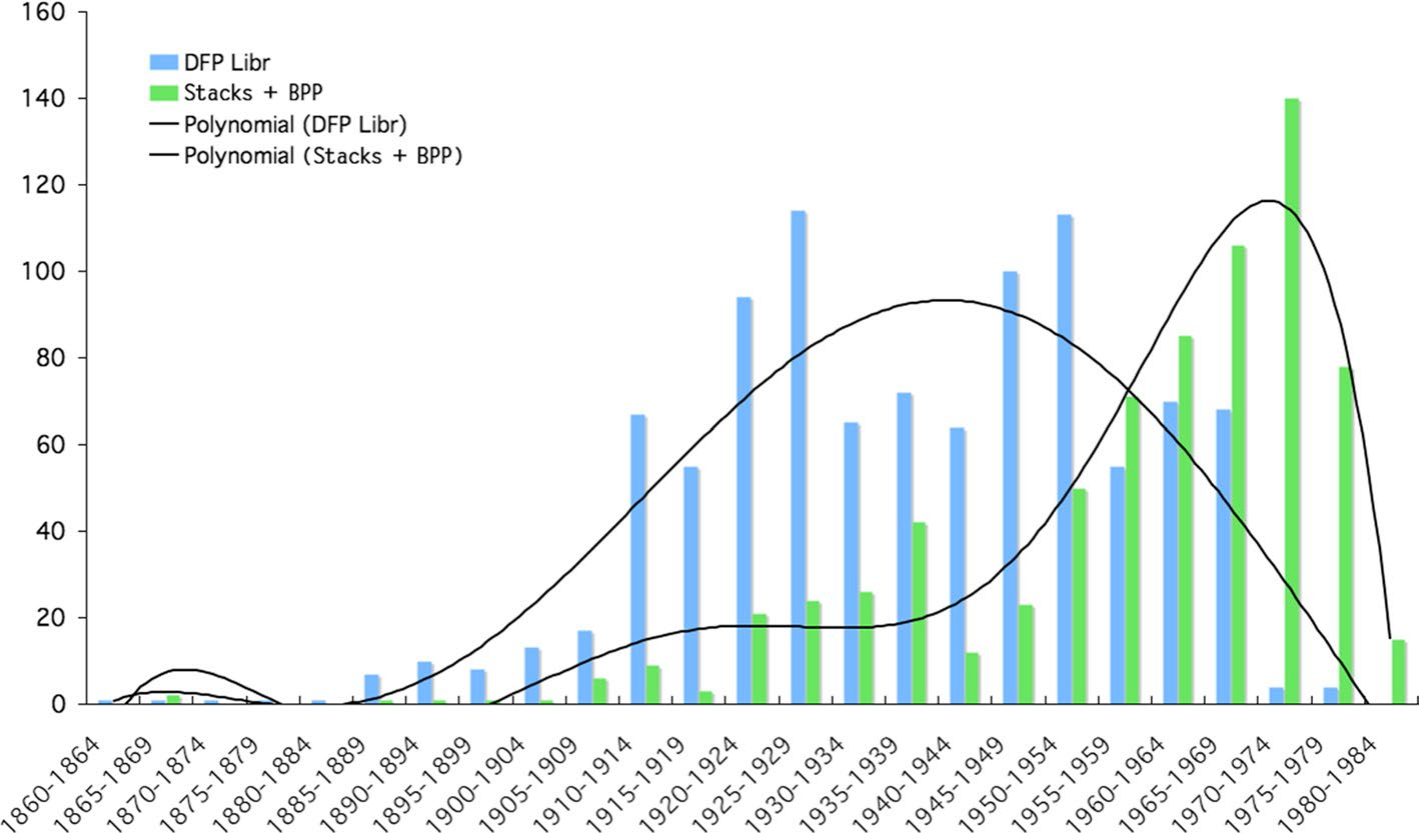
Frequency of books in the DFP library and in the BPP + stacks per five years

If we assume that horizontality was, from a time to be determined, the form taken by the segments left open by Piaget, we must try to understand the moment when the Archipilago was set up and the beginnings of the mobile office. The interest of the two donations separated in time (BPP and DFP) was that their contents and dates can be compared. We have already seen that one of Laurent’s sorting criteria was the horizontal versus vertical nature of the books, a characteristic that made them “librarianizable”. While the main operation was to group the stacked books for the first donation, he did not do so systematically, as some of the books on the large table to the right of the entrance, on the dresser, in the corners, in boxes under the tables, were not included in the first donation. The general question, however, is at what time did the closure of the wall-mounted libraries and the mobile office begin––mobility here in the sense of both moving the writing space and leaving part of the office in geological sedimentation. And thus creating the Archipilago system. To answer this question, we hypothesized that all remaining piles on or under the desks and tables postdated the starting of the mobile desk. A field in the database, spatial original information was used to make this grouping. We have therefore grouped together the “horizontal” books of the DFP found in 2012 with the BPP as opposed to the books vertically arranged in the shelves (from DFP) (Fig. 26).

The plot shows that the DFP library works (vertical books) extended from 1910 to 1960, while the archipilago works (horizontal books) extended from 1950 to 1980, with an intersection during the 1955–1959 period. These data suggest that the “closing” of the wall library and of the primitive desk occurred around the second half of the 1950s. What is fascinating here is that this date plays an important role in the very history of Piaget’s research culture: it corresponds to the period of opening and early development of the *Centre International d’Epistémologie Génétique* (Dionnet, 1998; Ratcliff & Tau, 2018; Burman, 2021), where Piaget was to introduce an interdisciplinary method for producing the Center’s results. This was a major change in his intellectual ecosystem, which led him to consider new ways of working. Therefore it is easy to understand why the mobile office and the archipilago were created aroud this time: faced with a change in the intellectual environment, linked both to the increase in data (books, files, manuscripts, letters) arriving at the house’s office, including the reconstruction of Europe––Piaget was also involved in IBE and UNESCO at this time (Hofstetter & Schneuwly, 2024)––and to a new way of organizing work at the Center, it seems that Piaget’s reaction was to *modify the shape* of this intellectual ecosystem, just as he showed molluscs and Sedum do when subjected to changes in their environment (Messerly, 2009: 96–97). The project management and the material environment thus interacted in his intellectual ecosystem, showing slow breaking points in response to a process of accumulation in the library (Cudré-Mauroux, 2018) that changed the order of the system.

## Conclusion

In conclusion, Jean Piaget’s library should be seen as part of an intellectual ecosystem. This system was made up of two fundamental elements that find coherency with the RBAB model: the archipilago, which was the mainly potential part, and the wall-mounted libraries, which became the closed part, around 1955.

This research used visual history, statistical analysis, and a behavioral model of the researcher dealing with his intellectual ecosystem to highlight the mechanisms of transformation that affected this environment. Within this framework, Piaget’s library was treated as part of an intellectual ecosystem, the analysis of which produced new results that either confirmed the achievements of Piagetian historiography (e.g., the interdisciplinarity reflected in his library, the relationship with disorder) or renewed it (e.g., the mobile office, his reading practices, the ranking of disciplines, and the temporality of the shift to the archipelago). Within a dynamic conception that the RBAB model has sought to understand, the office appeared as an organism in construction, animated by the researcher and subject to the multiple constraints of the environment. In this sense, it reflected something of Piaget’s theory of adaptation, in which the office became a kind of shell with differently organized, but above all evolving, temporalities and spaces. Through its mobility, Piaget’s office thus adapted to the quantitative and qualitative constraints that the academic environment gradually imposed on it. On this process of accumulation, Pruvost (2018) highlighted a property of libraries that “is coupled with a strange and disturbing vocation: to tend to expel the human beings who animate and frequent it”. It is something of this struggle that we coud see in Piaget’s mobile office history. A final way, materialized in this iconic place *par excellence* of Piagetian creation - Piaget’s office - to demonstrate the relevance of his theory.

## Data Availability

No datasets were generated during the current study.

## Footnotes

1 AJP « Convention de Donation » signed February 15, 2015.

2 AJP Cahiers des charges du secrétaire du Centre, 1967.

3 Personal communication, May 2009.

4 Archives nationales de France, 521 AP/57, Piaget to Ignace Meyerson, June 17, 1921.

## References

[CR1] Barbier, F. (2013). *Histoire des bibliothèques: d’Alexandrie aux bibliothèques virtuelles*. Colin.

[CR2] Belin, O. (2018). Paul Éluard: donner à lire. In O. Belin, C. Mayaux, & A. Verdure-Mary (Eds.), *Bibliothèques d’écrivains*. Rosenberg & Sellier. 10.4000/books.res.1991

[CR3] Belin, O., Mayaux, C., & Verdure-Mary, A. (Eds.) (2018). *Bibliothèques d’écrivains*. Rosenberg & Sellier. 10.4000/books.res.1721

[CR4] Bond, T., & Tryphon, A. (2009). Piaget and Method. In U. Müller, J. I. M. Carpendale, & L. Smith (Eds.), *The Cambridge companion to Piaget* (pp. 171–201). Cambridge University Press.

[CR5] Bringuier, J.-C. (1977). *Conversations libres avec Jean Piaget*. Laffont.

[CR6] Burman, J. T. (2016). *Constructive history: From the standard theory of developmental stages to Piaget’s new theory*. Doctoral dissertation, York University.

[CR7] Burman, J. T. (2021). *The genetic epistemology of Jean Piaget*. Oxford Research Encyclopedia of Psychology.

[CR8] Carbone, P. (2012). *Les bibliothèques*. PUF.

[CR9] Chapman, M. (1988). *Constructive evolution: Origins and development of Piaget’s thought*. Cambridge University Press.

[CR10] Chehab, M. (2018). La bibliothèque yourcenarienne: un creuset identitaire. In O. Belin, C. Mayaux, & A. Verdure-Mary (Eds.), *Bibliothèques d’écrivains*. Rosenberg & Sellier. 10.4000/books.res.1871

[CR11] Citko, H. (2018). La bibliothèque de Zbigniew Herbert (1924–1998). In O. Belin, C. Mayaux, & A. Verdure-Mary (Eds.), *Bibliothèques d’écrivains*. Rosenberg & Sellier. 10.4000/books.res.1836

[CR12] Cudré-Mauroux, S. (2018). La bibliothèque de Jean Starobinski, ce jardin ensauvagé. In O. Belin, C. Mayaux, & A. Verdure-Mary (Eds.), *Bibliothèques d’écrivains*. Rosenberg & Sellier. 10.4000/books.res.1826

[CR13] D’Iorio, P., & Ferrer, D. (Eds.). (2001). *Bibliothèques d’écrivains*. Editions du CNRS.

[CR14] Da Silva, D. L. (2023). Piaget-Wallon debate on the origin and development of symbolic thought. In R. H. De Freitas Campos, E. Lourenço, & M. J. Ratcliff (Eds.), *The transnational legacy of Jean Piaget. A view from the 21st century* (pp. 141–156). Springer.

[CR15] Dionnet, S. (1998). Production de connaissances et interaction: Le cas du Centre international d’épistémologie génétique. *Bulletin de psychologie*, *51*(3), 377–387.

[CR16] Ducret, J. J. (1990). *Jean Piaget: Biographie et parcours intellectuel*. Delachaux & Niestlé.

[CR17] Fumaroli, M. (2021). *Dans ma bibliothèque. La guerre et la paix*. Les Belles Lettres.

[CR18] García, R. (2000). *El conocimiento en construcción: De las formulaciones de Jean Piaget a la teoría de sistemas complejos*. Gedisa.

[CR19] Hofstetter, R., & Schneuwly, B. (2024). *The International Bureau of Education (1925–1968): The ascent from the individual to the Universal*. Macmillan.

[CR20] Jacob, C. (2001). Rassembler la mémoire. Réflexions sur l’histoire des bibliothèques. *Diogène*, *4*(196), 53–75.

[CR21] Jacob, C. (2014). *Qu’est-ce qu’un lieu de savoir?* OpenEdition.

[CR22] Kohler, R. (2008). *Jean Piaget*. Bloomsbury.

[CR23] Liengme Bessire, M. J., & Beguelin, S. (2008). Did Jean Piaget’s ‘conversion’ from malacology to psychology happen in the Faculty of arts? In A. N. Perret-Clermont, & J. M. Barrelet (Eds.), *Jean Piaget and Neuchâtel: The Learner and the Scholar* (pp. 62–74). Psychology Press.

[CR24] Messerly, J. G. (2009). Piaget’s biology. In U. Müller, J. I. M. Carpendale, & L. Smith (Eds.), *The Cambridge Companion to Piaget* (pp. 94–109). Cambridge University Press.

[CR25] Müller, U., Carpendale, J. I., & Smith, L. (Eds.). (2009). *The Cambridge companion to Piaget*. Cambridge University Press.

[CR26] Noël, A. I. (2020). *La construction et le contrôle de l’image publique de Jean Piaget entre 1945 et 1980: la contribution des médias comme vecteur d’une théorie*. Doctoral dissertation, University of Geneva.

[CR27] Ouvry-Vial, B. (2007). Valeur et enjeux fictionnels de la bibliothèque. *Questions de Communication, *hal-02264011.

[CR28] Parrat-Dayan, S. (1993). Le texte et ses voix: Piaget lu par ses pairs dans le milieu pédagogique des années 1920–1930. *Archives de psychologie*, *61*, 127–152.

[CR29] Perret-Clermont, A. N., & Barrelet, J. M. (2008). *Jean Piaget and Neuchâtel: The learner and the scholar*. Psychology Press.

[CR30] Piaget, J. (1918). *Recherche*. La Concorde.

[CR31] Piaget, J. (1937). *La Construction du réel chez l’enfant*. Delachaux et Niestlé.

[CR32] Piaget, J. (1959). Les modèles abstraits sont-ils opposés aux interprétations psycho-physiologiques dans l’explication en psychologie. *Bulletin de psychologie*, *13*, 7–13.

[CR33] Piaget, J. (1981). *Le possible et le nécessaire*. PUF, t. 1.

[CR34] Piaget, J. (1983). *Le possible et le nécessaire*. PUF, t. 2.

[CR35] Piaget, J., & García, R. (1983). *Psychogenèse et histoire des sciences*. Flammarion.

[CR36] Piaget, J., & García, R. (1987). *Vers une logique des significations*. Murionde.

[CR37] Pruvost, J. (2018). «Les» bibliothèques de Bernard Quemada… et de son élève. In O. Belin, C. Mayaux, & A. Verdure-Mary (Eds.), *Bibliothèques d’écrivains*. Rosenberg & Sellier. 10.4000/books.res.1786

[CR38] Quarto (2010). Autorenbibliotheken. *Quarto, Zeitschrift des Schweizerischen Literaturarchivs*, Special Issue, 30/31.

[CR39] Ratcliff, M. J. (2015). Archives des savoirs concurrentiels et comportement archivistique: le modèle C.R.C.A. In J.-F. Bert et M. J. Ratcliff (Eds.), *Frontières d’archives, recherche, mémoire, savoirs* (pp. 17-28). Edition des Archives contemporaines.

[CR40] Ratcliff, M. J. (2024). Origins, Trends and Perspectives of Historical-Epistemological Research on Piaget. *Integrative psychological and behavioral science*, *58*, 23–34. 10.1007/s12124-023-09796-7 PMC10904485 37495755

[CR41] Ratcliff, M. J., & Burman, J. T. (2017). The mobile frontiers of Piaget’s psychology. From academic tourism to interdisciplinary collaboration. *Estudios de Psicologia,* 1–33.

[CR42] Ratcliff, M. J., & Tau, R. (2018). A networking model. The case of the international center for genetic epistemology. *Estudos E Pesquisas em Psicologia,**18*(4), 1215–1238.

[CR43] Reichler, C. (2010). La bibliothèque de Jean Starobinski. *Bulletin du Cercle d’études Jean Starobinski*, *2010*, 12–17.

[CR44] Richelle, M. (2000). L’esprit piagétien et le renouveau de l’esprit. In O. Houdé et C. Meljac (dir.) *L’esprit piagétien*, (pp. 225–236). PUF.

[CR45] Smith, L. (2009). Piaget’s Developmental Epistemology. In U. Müller, J. I. M. Carpendale, & L. Smith (Eds.), *The Cambridge Companion to Piaget* (pp. 64–93). Cambridge University Press.

[CR46] Vidal, F. (1994). *Piaget before Piaget*. Harvard University Press.

